# New insights into immune cells in cancer immunotherapy: from epigenetic modification, metabolic modulation to cell communication

**DOI:** 10.1002/mco2.551

**Published:** 2024-05-23

**Authors:** Sha Qin, Bin Xie, Qingyi Wang, Rui Yang, Jingyue Sun, Chaotao Hu, Shuang Liu, Yongguang Tao, Desheng Xiao

**Affiliations:** ^1^ Department of Pathology Xiangya Hospital Central South University Changsha Hunan China; ^2^ Department of Pathology School of Basic Medical Science Xiangya School of Medicine Central South University Changsha Hunan China; ^3^ Regenerative Medicine, Medical School University of Chinese Academy of Sciences Beijing China; ^4^ Department of Oncology Institute of Medical Sciences National Clinical Research Center for Geriatric Disorders Xiangya Hospital Central South University Changsha, Hunan, China. University Changsha Hunan China; ^5^ NHC Key Laboratory of Carcinogenesis Cancer Research Institute and School of Basic Medicine Central South university Changsha Hunan China

**Keywords:** cancer immunotherapy, epigenetic modification, immune cells communication, metabolic modulation

## Abstract

Cancer is one of the leading causes of death worldwide, and more effective ways of attacking cancer are being sought. Cancer immunotherapy is a new and effective therapeutic method after surgery, radiotherapy, chemotherapy, and targeted therapy. Cancer immunotherapy aims to kill tumor cells by stimulating or rebuilding the body's immune system, with specific efficiency and high safety. However, only few tumor patients respond to immunotherapy and due to the complex and variable characters of cancer immune escape, the behavior and regulatory mechanisms of immune cells need to be deeply explored from more dimensions. Epigenetic modifications, metabolic modulation, and cell‐to‐cell communication are key factors in immune cell adaptation and response to the complex tumor microenvironment. They collectively determine the state and function of immune cells through modulating gene expression, changing in energy and nutrient demands. In addition, immune cells engage in complex communication networks with other immune components, which are mediated by exosomes, cytokines, and chemokines, and are pivotal in shaping the tumor progression and therapeutic response. Understanding the interactions and combined effects of such multidimensions mechanisms in immune cell modulation is important for revealing the mechanisms of immunotherapy failure and developing new therapeutic targets and strategies.

## Graphical abstract

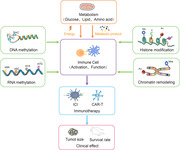

## 1 INTRODUCTION

**FIGURE 1:**
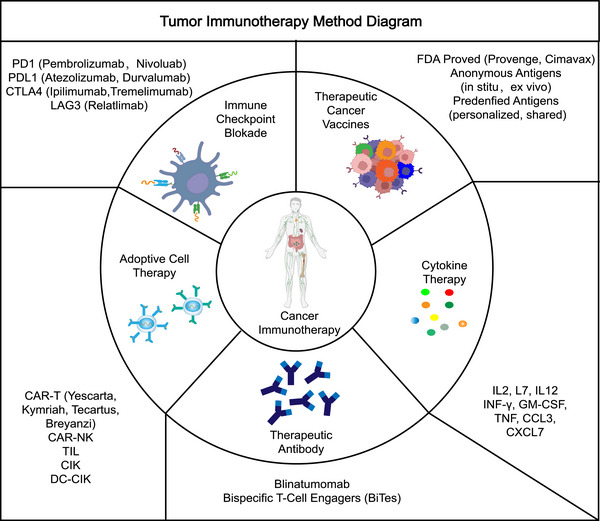
Principal tumor immunotherapy method. The tumor immunotherapy mainly including immune checkpoint blockades, therapeutic cancer vaccines, cytokine therapies, therapeutic antibodies, and adoptive cell therapies, which usually trigger T‐cell‐based immune responses against cancer antigens through the body's immune system, are now emerging as a unique pillar of cancer treatment.

Cancers are a challenge that has never been conquered, and they are a serious threat to human health. With regard to the treatment of cancers, surgery remains the first‐line treatment for many cancer types, and the clinical use of chemotherapy, radiation, neoadjuvant, and combination therapies have also improved patient survival, while the emergence of targeted therapies and immunotherapies may have the potential to revolutionize the treatment of cancer.^1^ Cancer immunotherapies (Figure 1), mainly including cytokine therapies, immune checkpoint blockades, therapeutic cancer vaccines, adoptive cell therapies, therapeutic antibodies such as bispecific T‐cell engagers, and so on, which usually trigger T‐cell‐based immune responses against cancer antigens through the body's immune system, are now emerging as a unique pillar of cancer treatment.^2^ However, the efficacy of cancer immunotherapy in a large part of patients is not satisfactory. The complexity of immune cells in recognizing and destroying cancer cells remains at the forefront of research, and the mechanisms are not well understood.

Epigenetic modifications are dynamically variable and they can alter the functions of nucleic acid and proteins on chromosomes through chemical modifications thus modulating genes expression at multiple levels, including splicing, transcription, translation, stability, and chromatin structure.^3^ Typical epigenetic modifications are DNA/RNA methylation, noncoding RNAs, histone modifications, and chromatin remodeling. Currently, there is evidence that epigenetic modifications play an essential role in the cancer immune response. For example, epigenetic regulation including DNA methylation, histone methylation, histone acetylation, and histone ubiquitination were proved to target programmed cell death protein 1 (PD1)/programmed cell death lidand 1 (PDL1), and these epigenetic regulations were found to be critical in the immunotherapy of malignant melanoma and hepatocellular carcinoma (HCC).^4^, ^5^ Other epigenetic modifications such as noncoding RNAs can also exert a regulatory role in the immune response by affecting immune checkpoints, epithelial–mesenchymal transition, the tumor immune microenvironment (TIME) including modulating the activation of immune cells such as T cells, dendritic cells (DC), myeloid‐derived suppressor cells (MDSC), and B cells.^6^, ^7^, ^8^ These evidences indicated that epigenetic mechanisms are critical in immune cell differentiation and function, ensuring that immune cells can exhibit specific gene expression patterns in different TIME conditions, thereby influencing the efficacy of cancer immunotherapy.

Tumor cells can modify metabolic pathways to enable them to adapt to the adverse microenvironment. In the tumor environment, the body often suffers from intensified glycolysis, enhanced lipid synthesis, altered amino acid metabolism, and higher production of lactic acid (LA), and these metabolic changes provide tumor cells with an advantage for proliferation and survival. And the metabolic competition that exists between the tumor and the immune cells hinders the function of the later.^9^ For example, prostate cancer cells release 1‐pyrroline‐5 carboxylate and repress T cell function to produce inflammatory cytokines through triggering of the shp 1/cytochrome *c* oxidoreductase/ROS axis.^10^ Furthermore, it has been shown that amino acid metabolism could strongly influence the tumor therapy resistance by controlling the destiny of diverse immune cells.^11^ That is to say, metabolism process in cancer cells and immune cells might be different, and the metabolism pathways in TIME are complexed and need to perform further researches.

Cell‐to‐cell communication within the TIME provides an important signaling for tumorigenesis and tumor development, and the investigation of message exchanges between different immune cells is crucial for the development of tumor immunotherapy and will help improve immune response rates.^12^ For example, DC‐based vaccines have proven to be one of the most promising therapeutic agents for cancer treatment. Researchers have found that enhancing the interaction between DCs and T cells by engineering DCs can facilitate tumor‐specific T‐cell activation and enhance immune response effects.^13^ So, targeting immune cell communication also is a great strategy for cancer immunotherapy.

Immunotherapy is based on the activation and functional execution of immune cells, and this process is regulated at multiple levels. The success of cancer immunotherapy not only relies on the direct activation of immune cells, but also on the comprehensive understanding and precise regulation of the complex behaviors and interactions of immune cells in the tumor microenvironment. Epigenetic modification, as an important mechanism for determining gene expression status, directly affects the differentiation and function of immune cells. The metabolic state of immune cells not only determines their energy supply, but also affects their response capacity and survival, especially in the immunosuppressed tumor microenvironment. Finally, cellular communication is critical for coordinating immune cell behavior and tumor cell interactions, affecting the efficiency of immune surveillance and tumor cell clearance. Taken together, the latest findings in these areas can provide a comprehensive perspective on the mechanisms of immune cell action in cancer therapy and offer important directions for overcoming the limitations of existing therapies and improving the efficacy of immunotherapy. We provide a relatively comprehensive review on the impact of cancer immunotherapy by focusing on immune cells from the perspective of their epigenetic modifications, metabolic reprogramming, and cell‐to‐cell communication. In particular, we provided some clinical trial results for cancer immunotherapy based on immune cell therapy and illustrated the relationship between immune cells and clinical prognosis, also summarized the currently identified biomarkers that can be used to assess and predict the cancer immunotherapy efficacy and briefly characterize frequent mouse models for investigation of cancer immunotherapy, which may provide new intensive research ideas and opportunities for further refinement of immunotherapies.

## 2 RELATIONSHIP BETWEEN IMMUNE CELL AND CANCER IMMUNOTHERAPY

The relationship between immune cells and the efficacy of cancer immunotherapy is complicated and multifaceted.^14^ The efficacy of cancer immunotherapy is influenced by a variety of elements, among which the number, activity, and functional status of immune cells and their interaction with tumor cells are crucial.^15^ Immune cells such as T cells, NK cells, and B cells are essential components of the body involved in the immune response, and their status and function directly affect the response of the immune system as well as the efficacy of immunotherapy.^16^, ^17^, ^18^

### 2.1 Effector T cells and cancer immunotherapy

The main purpose of immunotherapy is to enhance the body's immune response and one of the most common ways is to enhance T cell function, especially CD8+ T cells, so that they can more effectively recognize and eliminate cancer cells. There are many studies targeting effector T cells to enhance immune efficacy. CD8+ T cell phenotype is markedly increased after receptor activator of nuclear factor‐κB deletion, which enhances the efficacy of breast cancer immunotherapy.^19^ Metformin was found to improve the survival and the effector function of CD8+ T cells under hypoxic conditions as well as reduce hypoxia‐induced apoptosis, while inhibiting PD1 and LAG3, thus improving the efficacy of cancer immunotherapy.^20^ The stress response states (STRs), which is characterized as expressing heat shock genes, were significantly upregulated in immune checkpoint blockade treated unresponsive tumors, and T cells could link STR to immunotherapy resistance, suggesting that T cells may take a role in immunotherapeutic efficiency.^21^ Studies have demonstrated that the utilization of EP4 antagonist, YY001, enhances the proliferation and anticancer function of T cells while inhibiting the differentiation and maturation of MDSCs, thereby reversing the level of infiltration of MDSCs and T cells in the tumor microenvironment, and can be used for enhancing the prostate cancer immunotherapy efficiency.^22^ In conclusion, in many cases, effector T cells are a reflection of cancer immunotherapy efficiency. The number, function, and activation of effector T cells, represented by CD8+T, can often be used as an observable indicator for the efficacy of cancer immunotherapy.

### 2.2 Other T cells and cancer immunotherapy

In addition to effector T cells, other T cells are also critical for cancer immunotherapy efficacy. So far it has been discovered that tissue‐resident memory T cells are prognostic markers for a variety of cancers, including breast and lung cancers, and potential biomarkers for predicting the efficacy of immunotherapies such as immune checkpoint blockade and cancer vaccines.^23^ In addition, the tissue‐resident memory T cells are regulated by TGF‐β and they interact with tumor cells, DC cells, and other T cells in the TIME to ensure a positive T cell effector state.^24^ In addition, it has been demonstrated that neoantigen‐specific T cells have high affinity and perform an essential role in adoptive cell therapy (ACT).^25^, ^26^ Recent studies have revealed that T cells associated with recognizing cancer‐independent antigens (“bystander T cells”) also perform important functions in cancer immunotherapy and they differ in specificity, activation state, and effector function.^27^ Meanwhile, the exhausted T cells and immunosuppressive macrophages exhibited enhanced interactions in urothelial carcinoma of the bladder and performed significant functions in antitumor immunotherapy.^28^ Deletion of sorting nexin 9 (SNX9) in CD8+ T cells reduces the exhaustion of CD8+ T cells and enhances both T memory cell differentiation and interferon‐γ (IFNγ) secretion of adoptively transferred T cells, thereby enabling effective CAR‐T cells immunotherapy.^29^ In addition, the differentiation and modulation of T(H) subpopulations as well as γδ T cells in the TIME is critical for cancer immunotherapy efficacy.^30^, ^31^ Cellular senescence of tumor infiltrating lymphocytes (TIL) has recently been identified as an important state of T cell dysfunction in malignancy. Cancer cells and Treg cells are capable to induce senescence of responsive T cells through MAPK signaling, thus suppressing antitumor immunity and immunotherapy.^32^ In a word, besides effector T cells, tissue‐resident memory T cells, neoantigen‐specific T cells, “bystander T cells”, exhausted T cells, T memory cells, Th subpopulations, γδ T cells, senescence of TILs, and Treg cells also are significant index of cancer immunotherapy efficiency and worthy to be further studied.

### 2.3 Other immune cells and cancer immunotherapy

It is generally accepted that the eventual aim of cancer therapy is to induce a prolonged memory T cell response. So, the degree and function of T cell infiltration exerts an essential role in the response to tumor therapy. However, Chen et al.^33^ predicted the efficacy of anti‐PD1/PD‐L1 immunotherapy and survival rates in gastric cancer patients by multidimensional tumor‐infiltrating immune cells (TIICs) labeling. Also, Jiang et al.^34^ constructed a TIICs model for immune cell‐targeted therapy of gastric cancer and predicted the immunotherapy effect, which revealing that other immune cells in the TIME, such as B cells, macrophage cells, DC, and myeloid cells, also positively or negatively modulate the efficacy of antitumor immunity.

Here are some specific examples. B cells have been found to trigger antitumor immune responses either by direct tumor killing or by interacting with T cells and follicular DCs in tertiary lymphoid structures (TLSs). However, B cells can also acquire a suppressive function under certain conditions, becoming transformed into regulatory B cells that inhibit the tumor immune response.^35^ Depletion of tumor‐infiltrating B cells (TIBs) to enhance antitumor immunity is a novel and promising immunotherapeutic approach. Remodeling the TIME by decreasing the proportion of TIBs, enhancing the infiltration of CD8+/CD4+ T cells, and inhibiting the proliferation of Treg cells, as well as increasing the secretion of IL‐2 and IFN‐γ and decreasing the secretion of IL‐10, IL‐4, and TGF‐β, could improve cancer immunotherapy efficiency.^36^ Bufalin, as an antitumor immunomodulator, activates antitumor immune response by promoting the polarization of tumor‐infiltrating macrophages (TIMs) toward the M1 phenotype, and meanwhile facilitates the production of immunostimulatory cytokines by regulating the NF‐κB signaling pathway, thus effectively improving the therapeutic efficacy of HCC immunotherapy.^37^ Poly(I:C) and resiquimod combination therapy provides a synergistic activation of macrophage‐mediated antitumor immune response.^38^ DC vaccines induces specific CD8+/CD4+ T cell responses, and the DC‐cell‐based cancer immunotherapy possesses promising future perspectives.^39^ In addition, tumor‐infiltrating plasmacytoid DCs (pDCs) are known to have critical roles in the triggering and maintenance of antitumor immunity. The activated pDCs (IRF7+) were identified as a key regulator of adaptive antitumor immunity in CRC.^40^ In addition, TIL cells can also be used as markers to predict immunotherapy responsiveness and survival outcomes in melanoma,^26^, ^41^, ^42^ breast cancer,^43^, ^44^, ^45^ osteosarcoma,^46^ and other cancers.^47^, ^48^ All in all, current researches indicated that large amounts of immune cells possess the potential to affect cancer immunotherapy efficiency. And some of these immune cells such as different subset of B cells and T cells might exert extremely different effect (to be better or worser indicator for cancer immunotherapy efficiency), and these different isoforms can transform into each other under certain conditions.

Based on the important role of immune cells in tumor immunotherapy, we will next explore the function and mechanism of cancer immunomodulation in terms of epigenetics modification, metabolism modulation, and cell‐to‐cell communication in immune cells to provide new possible directions for tumor immunotherapy.

## 3 EPIGENETIC MODIFICATION

Epigenetics explores changes in the expression of genetic information that do not involve alterations in the DNA sequence, but rather chemical modifications that control the turning on and off of genes. The aim of this chapter is to review recent advances in epigenetic modifications in tumor cells and immune cells, focusing mainly on aspects related to DNA/RNA, histone, and chromatin modification.

### 3.1 Epigenetic modifications in tumors

Cancer is known to be a disease triggered and driven by genetic abnormalities, but epigenetic pathways also play an important role in tumorigenesis. And many features of cancer, including differentiation blockade, self‐renewal, cell death evasion, and invasiveness, are deeply affected by epigenomic changes.^49^ Epigenetic modifications have attracted increasing attention as anticancer strategies in recent years, which is largely based on their direct impact on cancer cells.

The epigenetic modification such as DNA and RNA methylation modifications have been shown to play an important role in tumorigenesis and diagnosis. For example, the researchers analyzed DNA methylation profiles in colon cancer and discovered that the levels of 5‐hmdC and 5‐mdC modifications were low, 5‐fdC modifications were the lowest, and 5‐cadC modifications were high, suggesting a relationship between aberrant patterns of DNA epigenetic modifications and cancer development.^50^ Liang et al.^51^ conducted DNA methylation analysis using high‐throughput DNA bisulfite sequencing of early‐stage lung cancer tissue samples and found that DNA methylation patterns were differentially expressed in tumors and benign lesions in circulating tumor DNA (ctDNA). In addition, deficiency of ALKBH5‐mediated m6A modification in osteosarcoma causes an increase expression of histone deubiquitinase USP22 and ubiquitin ligase RNF40, which inhibits histone H2AK199 monoubiquitination, induces the expression of genes related to DNA repair, and promotes the progression of osteosarcoma.^52^ The m6A RNA methylation in LINC01559 was found to inhibit the progression of colorectal cancer (CRC) by modulating the miR‐106b‐5p/PTEN axis.^53^ The m6A, m5C, m7G, and m1A‐related genes have also been demonstrated to have a correlation with immunotherapy response in cervical cancer,^54^ HCC,^55^ and glioma.^56^ However, the mechanisms and exact functions of these modifications in cancer still need further investigation.

In addition, histone modifications are another common epigenetic mark that involves the maintenance of stability of chromatin, as well as dynamic cellular processes like transcription and DNA repair.^57^ A recent research demonstrated that the lactonization of H3 histone modification sites H3K9la and H3K56la promoted the progression of HCC.^58^ In addition, H3K14, H4K5, and H4K12 histone acetylation promote non‐small cell lung carcinoma (NSCLC) cell growth.^59^ Different modifications of histones can be mutually modulated. For example, the deacetylation of AKAP12 at K531 by HDAC6 can increase its level of ubiquitination, promotes the proteasome‐dependent degradation of AKAP12 and facilitates colon cancer metastasis.^60^ H3K27 acetylation‐activated COL6A1 inhibits STAT1 by interacting with SOCS5 in a ubiquitinated and proteasomal degradation manner thereby promoting osteosarcoma progression.^61^ Inhibition of histone deacetylase protein 6 (HDAC6) decreases ERK phosphorylation levels and inhibits cancer proliferation by upregulating microtubule protein acetylation.^62^ Histone methyltransferase DOT1L affects prostate cancer progression by increasing the methylation at H3K79 of MYC and decreasing expression of E3 ubiquitin ligases HECTD4 and MYCBP2.^63^ The methyltransferase SMYD3 mediates H3K4 trimethylation at the cold shock structural domain‐containing protein E1 (CSDE1) and contributes to STAT1 dephosphorylation through stabilization of the T cell protein tyrosine phosphatase, which alters the antigenicity of tumor cells.^64^ The methyltransferases SMYD3 promotes RNF113A K20me3 methylation modification while impairing the interaction with phosphatase PP4, which downregulates its phosphorylation level, promotes and maintains RNF113A E3 ligase activity, and inhibits chemo‐sensitivity in small cell lung cancer.^65^ In a word, histone modifications act as a part of epigenetic area, they could participate in cancer progress alone or together with several different histone modifications form. Also, some epigenetic modifications among cancer seem to be involved in immune cells or immunotherapy. As the epigenetic modifications and immunotherapy are believed to be hot pot in recent cancer researches. So, it is urgent to figure out the specific mechanism of epigenetic modifications in immune modulation.

### 3.2 Epigenetic modifications of immune cells in the TIME

In recent years, the contribution of the immune system in restraining the development and progression of cancer has been largely acknowledged. New evidence is emerging that tumors usually hijack diverse epigenetic mechanisms to evade immune system constraints, and the immune cells engaged in antitumor response also may be influenced by altered epigenomes.^66^

#### 3.2.1 DNA/RNA methylation modifications of immune cells in TIME

Epigenomic profiles of immune cells can serve as potential predictors for the evaluation of tumor immunotherapy. In addition, since epigenetic modifications are dynamic, they are expected to be targets for modulating immunotherapy.^67^ DNA methylation, as a kind of typical epigenetic modifications, has been shown to mold the epigenetic landscape of CD8+ T cells transfer to exhausted T cells or T memory cells, which can be persistent over time.^68^ It has been shown that treatment with demethylating agents recovered the immunogenicity of cancer cells, while enhancing the capacity of CD8+ T cells to kill tumors.^69^, ^70^ Additionally, RNA methylation can exert influence on immune cell maturation and function to modulate tumor immunity in the TIME. For instance, the deficiency of METTL3 (m6A “writer” protein) in T cells destroys T cell homeostasis and differentiation by targeting the IL‐7/STAT5/SOCS pathway. That is because deficiency m6A modification in T cells inhibits SOCS mRNA degradation, which in turn inhibits IL‐7‐mediated STAT5 activation and also inhibits the T cell homeostasis and differentiation.^71^ In addition, it was found that macrophage‐specific knockdown of the m6A methyltransferase Mettl14 resulted in elevated expression levels of the cytokine subunit EBI3, which could lead to dysfunction of CD8+ T cells, thereby impairing its tumor elimination ability.^72^ In conclusion, methylation modifications had a potential to control immune cell phenotype (such as T cells), immune cell maturation and function, and modulating immune‐related pathways.

#### 3.2.2 Histone modifications of immune cells in TIME

The effect of epigenetic modifications of histones in TIME should not be ignored. Epigenetic modulators have also been proven to be critical in controlling macrophage fate. And the histone‐modifying enzymes usually work together with some transcription factors to regulate gene activity and perform essential roles in the development of antitumor immunity. For example, HDAC2 modulates the M2‐like TAM phenotype through acetylation of the transcription factor SP1 and histone H3. The deletion of HDAC2 induced the transition from M2 to M1‐like TAM, activated T‐cell activation‐related signaling pathways, reduced CD4+ T cells and increased CD8+ T‐cell infiltration, and inhibited the lung cancer progression.^73^ Furthermore, downregulation of HDAC8 increased H3K27 acetylation, increased CD8+ T cell expression, and reactivated T cells to produce chemokines, thereby enhancing the immunotherapeutic efficacy of HCC.^74^ In addition, ketolysis can also alter the CD8+ T cell function by modulating histone acetylation.^75^ Inhibition of HDAC1 and HDAC2 also induced the expression of natural killer (NK) G2D (NKG2D) ligand and enhanced NK cell‐mediated anticancer immunity.^76^ HDAC3 can modify H3K27ac of Cxcl10 promoter. And HDAC3‐deficient HCC cells recruited CXCR3+ T cells into the tumor microenvironment to inhibit tumor growth.^77^ In addition, it was demonstrated that reduced epigenetic histone modification (such as H3K4me1, H3K9me2, H3S10p, and H2BK16ac) are associated with the death of mast cell HMC‐1, providing new ideas for the pathogenesis of mast cell leukemia.^78^ In conclusion, histone modifications, especially HDAC‐based deacetylation modifications, regulate immune cell differentiation and function. Notably, KAT6A acetylation of SMAD3 regulates MDSC recruitment and immunotherapy in triple‐negative breast cancer (TNBC), providing new insights into the targeting of epigenetic factors in immunotherapy to enhance the therapeutic efficacy.^79^ This indicates that some nonhistone acetylation modifications also regulate immune cell fate.

#### 3.2.3 Other chromosome modifications of immune cells in TIME

##### Polycomb family

The Polycomb family (PcG) is a group of highly conserved chromatin modifiers, mainly comprising Polycomb repressive complex 2 (PRC2), a methyltransferase for histone H3K27 methylation, and PRC1, which has E3 ubiquitin ligase activity.^80^ Members of PRC1 include the CBX family and the PHC family, while members of PRC2 include the EZH family, SUZ12, and EED. This family mainly works by modifying chromatin structure in order to maintain the silenced state of genes.

The PcG has been discovered to participate in modulating immune cell fate. CBX7 can activate CD4+ T cell polarization and apoptosis by regulating FasL expression.^81^ CBX4 deficiency leads to reduced accumulation of inhibitory histone modifications such as H2AK119ub1 and H3K27me3 of the PDCD1 promoter in T cells, and thus inhibiting antitumor immunity.^82^ In addition, the role of PRC2 member in immune cells also been researched. Inhibition of EZH1 can epigenetically remodel iPSCs to efficiently generate mature T cells for immunotherapy.^83^ Inhibition of EZH2 reduces antitumor immune efficiency by reprogramming CD8+ T cells and reducing their survival and effector functions.^84^ In bladder cancer, inhibition of EZH2 induces NK cell‐mediated immune responses leading to tumor cell differentiation and death.^85^ Inhibition of EZH2 stimulated the production of chemokines such as CCL2 and CXCL9/10, leading to NK cell and T cell infiltration in pancreatic ductal adenocarcinoma.^86^ EZH2 maintains silencing on the FOXP3 gene through H3K27me3 labeling, which prevents overactivation of T cells and helps maintain immune tolerance.^87^ Similarly, EZH2 has been identified to inhibit NK cell proliferation and promote the progress of cancer cells through modulating the H3K27 methylation and downregulate NKG2D ligands.^88^ Also, EZH2 is involved in the regulation of epigenetic reprogramming in Treg cells, which resulted in a proinflammatory function and maintains the survival and function of effector CD4+/CD8 T cells through remodeling TIME.^89^ Currently, most studies on the effects of PcG members on immune cells have focused on PRC2, especially EZH2, while studies on PRC1 members such as the PHC family are almost absent.

##### CTCF

**FIGURE 2:**
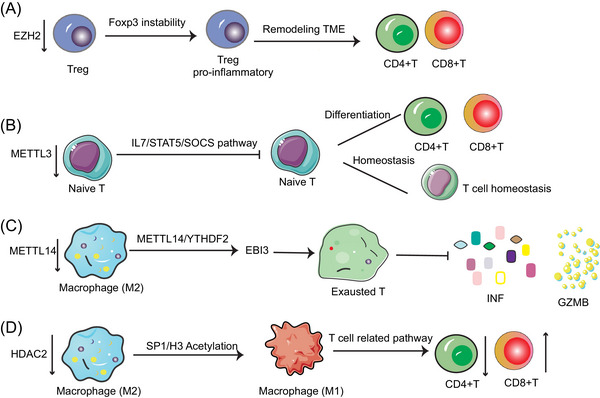
Epigenetic modifications in immune cells. (A) Downregulation of EZH2 in Treg cell leads to Foxp3 instability, causing an increase of proinflammatory Treg cell numbers and leading to an increase in CD4+T and CD8+T through remodeling of the TIME. (B) Downregulation of METTL3 inhibits the differentiation and cell homeostasis of Naive T cells through the IL7/STAT5/SOCS pathway. (C) Downregulation of METTL14 in macrophage (M2) promotes EBI3 expression in an m6A‐dependent manner, contributes to T cell exhaustion, and inhibits INF and GZMB production. (D) Downregulation of HDAC2 in macrophage (M2) regulates macrophage polarization via transcription factors SP1 and H3 histone acetylation modification, and M1 macrophage inhibits CD4+ T‐cell function while promoting CD8+ T function via T cell‐related pathways. (TIME, tumor immune microenvironment; INF, interferon; GZMB, granzyme B).

In addition, some transcription factors bind to DNA to ensure precise regulation of gene expression and maintenance of structural stability of chromatin. They are critical for biological processes such as cell development and differentiation. CTCF, a DNA‐binding protein involved in the formation of chromatin circuits, which has two main functions, one is to act as a chromatin terminator, preventing chromatin regions from interacting, and the other is to act as a medium of chromatin interconnection, facilitating chromatin regions to interact.^90^, ^91^, ^92^ It has been shown that Tcf1 recruits CTCF and promotes chromatin interactions to regulate the genomic structure and homeostatic proliferation of CD8+ T cells.^93^ CTCF regulates CD8+ T cell heterogeneity and promotes terminal differentiation of CD8+ T cells by altering the transcription factor landscape and transcriptome interactions.^94^ CTCF is required to establish NF‐κB‐dependent chromatin interactions in DCs to promote inflammatory response.^95^ However, another research declaims that CTCF is dispensable for immune cell trans‐differentiation although it affects the topologically associating domains but rather facilitates rapid responses to external stimuli.^96^ All in all, CTCF is involved in the regulation of gene expression in immune cells by modulating chromatin structure to ensure the differentiation and function of immune cells. Investigating the function of CTCF can help reveal the epigenetic regulatory mechanisms of immune cells and provide new targets and strategies for the cancer immunotherapy treatment. In conclusion, epigenetic modifications can modulate tumor immunity by regulating the function of multiple immune cell subtypes and can also reshape the immune response by regulating the expression of immune‐related genes (Figure 2). Consequently, specific reprogramming through epigenetic modification of immune cells holds great promise for remodeling aspects of tumor microenvironment. Epigenetic modifications are largely dependent on various enzymes, and specific functions of epigenetic regulation are investigated by knocking down these enzymes. A number of drugs targeting these enzymes have also been developed, such as targeting DNA methyltransferases (e.g., 5‐azacytidine, decitabine, and guadecitabine), EZH2 inhibitors (e.g., Tazemetostat and GSK126), HDAC inhibitors (e.g., vorinostat, romidepsin, and panobinostat) making research more convenient and laying the foundation for clinical translation of epigenetic modifications.

## 4 METABOLIC MODULATION

Metabolic processes are fundamental to life activities and are involved in all chemical reactions throughout an organism, supporting cell growth, division, and energy production. Modulating the activity and direction of metabolic pathways can affect the state and function of cells and organisms. The aim of this section is to explore the recent discoveries in metabolic modifications and their role in tumor and immune cell function and tumor progression.

### 4.1 Metabolic reprogramming in tumors

With a more sophisticated understanding of tumor biology and the complexity of tumor metabolism, it is now believed that the metabolic reprogramming is one of the hallmarks of cancer.^97^ There is no doubt that cancer cells have different metabolic states compared to normal cells, and cancer cells can show great metabolic diversity and plasticity in different tumors.^98^ Studies have shown that metabolic phenotypes vary at different stages of cancer development. For example, tumors need more nutrients to grow in the early stages, while during metastasis stage, new metabolic phenotypic dependencies often emerge due to the treatment resistance in tumors.^99^ The metabolic resources within local tumor tissues are limited, creating a tumor microenvironment of nutrient depletion and metabolic waste accumulation. In order to maintain growth under various conditions of nutrient distribution in the body, cancer cells utilize different metabolic adaptations. And the metabolites of cancer cells not only can be used as substrates for a source of energy, but also can adjust the expression of genes and proteins in normal cells so as to influence their behaviors.^100^ Luo et al.^101^ analyzed the metabolic phenotype of HCC cells and found that the expression of CD36 was positively correlated with the degree of glycolysis. Overexpression of CD36 induced an increase in mammalian target of rapamycin (mTOR)‐mediated glycolytic flux and lactate generation through activation of the Src/PI3K/AKT signaling pathway, thereby promoting the progression of HCC.^101^ In non‐small cell lung cancer, LINE‐1 is activated by L1 antisense promoter (L1‐ asp) or reverse transcription to form L1 gene chimeric transcript, which participates in the metabolic process of tumors and the regulation of mitochondrial function, facilitating lung cancer progression.^102^ In addition, a strong link exists between tumor metabolism and immune cell metabolism, as metabolic changes in the TIME can affect immune cell function and response.

### 4.2 Metabolic modulation of immune cells

#### 4.2.1 Influence of metabolism and its products on immune cell phenotype in the TIME

Cellular metabolism governs the differentiation of immune cells and regulates their function, which is one of the fundamental processes that sustains cellular life and is a critical driving force in determining cell fate.^103^ Since the tumor is a highly heterogeneous environment, each cell in the tumor exists in a different microenvironment, and each cell could also have its own metabolic state. The current understanding of the metabolic characteristics of immune cells has been inspired by the metabolism of cancer cells, though there are obvious discrepancies between cancer cell and immune cell metabolic reprogramming. Studies have found that the overall activity of metabolic pathways in tumor cells is higher and more plastic than in nontumor cells such as immune cells, which is more likely to lead to cell‐specific metabolic reprogramming in the tumor microenvironment.^104^, ^105^ And the variation of mitochondrial activity is the leading cause of metabolic heterogeneity between tumor cells and nontumor cells. Moreover, these differences may provide opportunities to target immune cell metabolism as a tool to enhance the efficacy of immunotherapies.^106^ Interactions during metabolic reprogramming of cancer cells and immune cells are thought to be key determinants of antitumor immune responses to cancer. Among these, tumor metabolism can modulate antitumor immune responses by releasing metabolites and influencing the expression of immune molecules (e.g., lactate.), and in turn, the metabolic reprogramming of various types of immune cells is also essential in maintaining their own and the organism's homeostasis.^9^ Immune cells are able to recognize diverse signals of tissue metabolism (e.g., nutrient and oxygen consumption, and reactive nitrogen production) in the microenvironment and initiate specific immune functions.^107^, ^108^ Metabolites also contribute to the phenotypic transformation of immune cells. It has been shown that B‐cell‐derived metabolites and the neurotransmitter GABA promote the differentiation of monocytes into anti‐inflammatory macrophages, which produce IL‐10 and suppress CD8+ T‐cell killing function, thereby inhibiting the antitumor response.^109^ Imbalances in the distribution of applicable nutrients, substrates, or other sources in immune cells can affect the metabolism of immune cells, and hence the function and destiny of the cells. What is more important is that whether metabolic changes are responsive or instructive throughout functional changes in immune cells is now still required more detailed researches.

#### 4.2.2 Glucose metabolism

Glucose metabolism has always been the core of the cancer metabolism field, yet recent studies have demonstrated that glucose in the TIME is preferentially assigned to infiltrating immune cells rather than to cancer cells, which reveals an important function of immune cell glucose metabolism.^110^ Studies have shown that the LA in TIME suppresses immune cell function and leads to immune escape. And the high glucose induces a Warburg effect in γδ T cells, leading to lactate accumulation, which in turn leads to the loss of antitumor capacity in patients by inhibiting the activation of the AMPK pathway.^111^ In addition, recent research has assumed that the alterations in the metabolic landscape of TIME are associated with increased activity of Treg cells. Treg cells have been found to upregulate the metabolic pathway of LA to produce intermediates required for proliferation and maintain an inhibitory identity by exploiting metabolites in the TIME.^112^ In highly glycolytic tumors (e.g., HCCs), Treg cells take up LA via monocarboxylate transporter 1 (MCT1), which facilitates the entry of NFAT1 into the nucleus and thus increases PD1 expression, whereas effector T cells inhibit PD1 expression, resulting in a higher level of PD1 expression in Treg cells than in effector T cells.^113^ MCT leads to the establishment of an extracellular acidic TIME that facilitates the selection of more invasive tumor cells and suppresses T cell mediated antitumor immune responses to promote tumor progression.^112^ In addition, metabolic reprogramming of NK cells maintains their metabolic adaptation in the tumor state and also enhances their tumor‐killing ability in the tumor microenvironment. In the TIME, the dysfunction of NK cells is mainly due to the inhibition of glucose metabolism by lipid peroxidation‐related oxidative stress, whereas the function of NK cells can be recovered by activation of the Nrf2 antioxidant pathway and leads to stronger antitumor activity.^114^ Yet another study showed that LA was able to enhance antitumor immunity by suppressing histone deacetylase activity, leading to an increase in acetylation of H3K27 at the Tcf7 super enhancer site, thereby increasing the stemness of CD8+ T cells. Meanwhile, CD8+ T cells pretreated with LA in vitro were capable of effectively suppressing tumor growth.^115^ These researches revealed that glucose metabolism especially LA affect the immune cell function through MCT, signaling pathways or altering the epigenetic modification to modulating cancer progression.

#### 4.2.3 Lipid metabolism

Fatty acids provide immune cells with cell membranes and other key lipid cell structures required for proliferation, and they can also be directly involved in signaling modulation within immune cells. For example, researchers have found that pentanoate and butanoate (short‐chain fatty acids) regulated the metabolic (mTOR pathway) and epigenetic reprogramming (modulation of histone deacetylase activity), leading to elevated production of effector molecules such as CD25, IFN‐γ, and TNF‐α to enhance the antitumor activity of CAR‐T cells.^116^ In addition, PGE2, a major metabolite produced from arachidonic acid, exerts an anti‐inflammatory effect on natural immune cells such as NK cells, neutrophils, and monocytes. It can also participate in immune responses as an immunosuppressive factor.^117^, ^118^, ^119^ For instance, PGE2‐EP2/EP4 signaling was proved to promote active inflammation by inducing NF‐κB gene expression in myeloid cells and to trigger immunosuppression by activating mregDC (mature DCs enriched in immunoregulatory molecules) and Treg cells.^120^ In addition, PGE2 was reported to promote the transformation of M1 macrophages into M2 macrophages through cAMP/cAMP‐responsive element binding signaling pathway.^121^ Cheng et al.^122^ revealed that there was aberrant lipid metabolism in HCC tissues by lipidomic analysis. And accumulation of long‐chain acylcarnitines, such as palmitoylcarnitine and stearoylcarnitine, suppressed NKT cell expansion and facilitated their senescence, which weakened their immune surveillance ability against tumors.^122^ Research demonstrated that T cell functional status is a critical determinant of effective antitumor immunity and immunotherapy. And the lipid metabolism has been reported to govern T cell differentiation and effector functions. For example, suppression of lipid metabolizing enzymes (IVA phospholipase A2) reprogrammed effector T cells and thus blocking CD8+ T‐cell senescence and boosts antitumor immunity and immunotherapeutic effects in melanoma and breast cancer.^123^ In conclusion, the effect of lipid metabolism on immune cells in tumors is a complex and dynamic process. Lipids maintain the energy balance of immune cells, modulate their polarization state and function, and lead to aberrant activation or inhibition of immune cell signaling pathways, thereby affecting their response to tumors. An in‐depth understanding of these interactions can help develop more targeted immunotherapy.

#### 4.2.4 Amino acid metabolism

Competition for amino acid metabolism also occurs in the tumor microenvironment. It was believed that increasing the level of amino acids in TIME was beneficial in enhancing the killing effect on immune cells. For example, glutamine is competitively consumed by effector T cells and TNBC cells, whereas specific blockade of glutamine uptake by TNBC cells using V‐9302 (glutamine transporter protein inhibitor) drives glutathione synthesis and improves effector function of CD8+ T cells.^124^ In addition, obstruction of glutamine metabolism has been shown to enhance anticancer immunity. Huang et al.^125^ demonstrated that JHU083 (a glutamine antagonist) increased the infiltration of CD8+ T cells and CD4+ Th1 cells and decreased immunosuppressive cells, such as MDSC, Treg cells, resulting in strengthened immunoprevention of lung cancer. Additionally, blocking glutamine induced different metabolic processes could prevent tumor cells to immune escape. Powell et al.^126^ discovered that glutamine blockade inhibits oxidative and glycolytic metabolism in cancer cells, which leads to hypoxia, acidosis, and decreased nutrient consumption, while with the use of glutamine antagonists, effector T cell oxidative metabolism was significantly upregulated with a significantly activated T cell phenotype. Additionally, arginine metabolism plays a crucial role in T cell activation and regulation of immune responses. It sensed by BAZ1B, PSIP1, and TSN and could modulate metabolic processes from glycolysis to oxidative phosphorylation in activated T cells and promote the production of T memory cells.^127^ So, arginine supplementation and prevention of arginine degradation in TIME are promising approaches to activate T cells to trigger positive immune response. Similarly, the activation of T cells is critically sensitive to the concentration of tryptophan in the TIME, which is consumed by the metabolism process of tumor cells, triggering T cell apoptosis and inhibiting the tumor‐killing function of T cells.^128^ Metabolic reprogramming of tumor methionine metabolism has been shown to be a potentially viable therapeutic strategy to enhance immunity in HCC. Hung et al.^129^ used the metabolomics analyses of HCC to demonstrate that elevation of 5‐methylthioadenosine and s‐adenosylmethionine induced T‐cell exhaustion by reducing chromatin accessibility and thus promoted the HCC progression. In conclusion, amino acid metabolism plays a key role in tumor immunity. Arginine and glutamine are important substrates that influence the activity of macrophages, MDSC, NK cells, and T cells, whereas tumor cells may influence immune cell function by competing for and limiting amino acid supply.

#### 4.2.5 Microbiome

The contribution of microbes in cancer development, diagnosis, prognosis, and therapy has received much attention in recent years. According to recent studies, microbes may be a key factor in cancer immunotherapy. The microbes in cancer cells and immune cells may be of the same origin. The microbes and their bioactive metabolites can be involved in immune regulatory processes in the development of tumors. Thus, there may be a common evolutionary dynamic between the host immune system, the microbiota, and tumor development.^130^ The researchers discovered that DCs from germ‐free animals and those without IFN‐I signaling had low levels of gene expression for genes involved in the mitochondrial respiratory chain. And further analysis demonstrated that the metabolism of the DCs cells from germ‐free animals was abnormal and they were unable to initiate immune responses, which suggests that the microbiome plays a crucial role in the function of DCs.^131^ Studies have demonstrated a link between bile acid metabolism controlled by the gut microbes and liver antitumor immune surveillance. The intestinal microbes promote TLR7–MyD88 signaling in pDCs through secondary bile acid metabolism, and promote IFN production.^132^ In addition, Ma et al.^133^ found that gut microbes regulate CXCL16 expression through bile acid metabolism, which in turn modulates an increase in NKT cells, leading to an increase in IFN‐γ production. Exposure to helicobacter hepaticus in a mouse model of CRC induced upregulation of specific T follicular helper cells (Tfh) and led to the maturation of tertiary lymphoid‐like structures adjacent to the tumor, which inhibited tumor growth. This study demonstrates that antitumor immunity is not dependent on CD8+ T cells but on CD4+ T cells, NK cells, as well as B cells.^134^ Microbiome deficiency slowed melanoma‐induced migration of intestinal NK cells and Th1 cells from the gut to the bone, thereby accelerating melanoma metastasis and intraosseous tumor growth.^135^ It was found that butyrate, a gut microbial metabolite, could contribute to the therapeutic efficacy of oxaliplatin by promoting the IL‐12 signaling pathway, which enhanced the response of antitumor cytotoxic CD8+ T cells in an ID2‐E47 dependent way.^136^ Peng et al.^137^ performed an analysis of the tumor microbiome of 53 gastric cancer patients and the gastric mucosal tissue microbiota of 30 chronic gastritis patients, revealing that Methylobacterium could reduce the expression of TGFβ and CD8+ T memory cells in tumors, and significantly correlate with the adverse prognosis of gastric cancer. It was found that deletion of the TAK1, an innate and adaptive immune molecule in myeloid cells, promotes the IL‐1β and IL‐6 signaling pathways (necessary for induction of Th17 cells) through the microbiome (Odoribacter splanchnicus), thus resulting in anti‐CRC properties.^138^ Thus, the microbiome is also critical for the functional modulation of immune cells, and targeting the microbiome is a potential direction for cancer immunotherapy.

#### 4.2.6 Relationship between mitochondrial function and the phenotype of immune cells

The membrane of mitochondria contains a large number of signaling molecules, which are important hubs for determining the fate of cells, and they serve important regulatory functions in cell signaling pathways and metabolism. It has been well established that mitochondria exert an essential driving role in the development and function of immune cells. For example, mitochondrial metabolism supports T cell anabolism by modulating key metabolic pathway cGAS–STING signal that control the fate and function of Treg cells.^139^ It has been shown that alterations in mitochondrial metabolism (involving oxidative metabolism, and the tricarboxylic acid cycle, mitochondrial membrane potential, reactive oxygen species (mtROS), DNA, and ultrastructure) may be the basis for macrophage activation, and that mitochondria may act as dynamic signals to modulate the immune response through modulation of macrophage biology.^140^ It was demonstrated that nanotubes transferred mitochondria from immune cells to cancer cells, which enhanced cancer cell metabolism and exhausted a large number of immune cells, while inhibiting the assembly of nanotubes greatly reduced the transfer of mitochondria and minimized the exhaustion of immune cells.^141^ Additionally, inhibition of the mitochondrial membrane protein FAM73b promotes Toll‐like receptor‐regulated (TLR)‐mediated mitochondrial fission and induces IL12 production through stabilizing IRF1 in macrophages, thereby promoting CD8+ T cell activation to antitumor immunity.^142^ As an important hub for determining cell fate, mitochondria possess modulatory functions in cell signaling pathways and energy metabolism. The role of mitochondrial function in tumor cell and immune cell metabolism and dysfunction is a challenge in cancer immunometabolism research and might be a promising research area for cancer immunotherapy in the future.

#### 4.2.7 Relationship between ions metabolic pathways and immune cells

**FIGURE 3:**
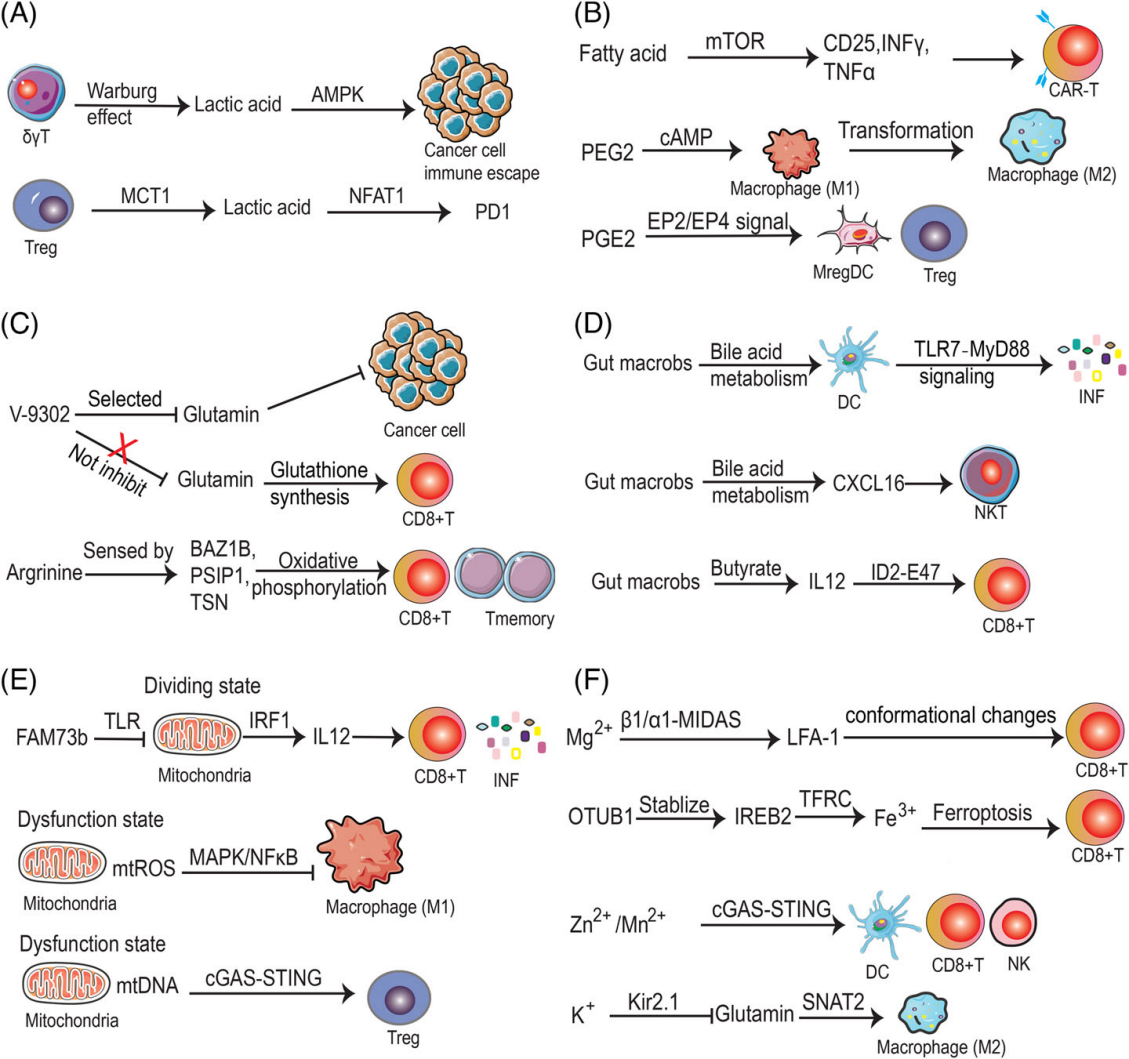
Metabolic modulation of immune cells. (A) Glucose metabolism. Lactate production by δγT and Treg cells via glycolysis facilitates tumor immune escape. (B) Lipid metabolism. Fatty acid metabolism enhances CAR‐T cell function and PEG2 promotes M2, Treg, and MregDC function. (C) Amino acid metabolism. Glutamate and arginine promote effector T cell function by increasing glutathione synthesis and oxidative phosphorylation. (D) Microbiome and immune cell metabolism. The microbiome regulates NKT, DC, and CD8+T functions through bile acid metabolism and butyrate. (E) mitochondrial function and the phenotype of immune cells. Dividing state mitochondria promote CD8+ T phenotypes. Mitochondrial dysfunction can lead to immunosuppression through NFκB and cGAS–STING signaling. (F) Ions metabolic pathways and immune cells. Mg^2+^, Fe^3+^, Zn^2+^, Mn^2+^ promote effector T cell function, and K^+^ could also promotes M2 macrophage function.

In recent years, multiple ion metabolisms have been found to be involved in modulating cancer immunity. For example, magnesium ions could induce conformational changes of LFA‐1 by binding to β1‐MIDAS and α1‐MIDAS on the membrane of CD8+ T cells, thus altering their cytotoxicity and promoting tumor cell killing functions.^143^ OTUD1 promotes the deubiquitination of IREB2, which inhibits the degradation of IREB2 protein and activates the expression of TFRC, thereby increasing the concentration of iron ions in tumor cells and enhancing the sensitivity to ferroptosis as well as the infiltration of CD8+ T cells, improving antitumor immune response.^144^ In addition, high potassium ions concentrations in tumors disrupt glutamine uptake and reprogram TAM metabolism from oxidative phosphorylation to glycolysis, thereby inhibiting the antitumor capacity of TAM.^145^ Studies have revealed that Mn^2+^ can effectively activate the cGAS–STING pathway, which significantly promotes the function of DC and enhances the survival, proliferation, and function of cytotoxic T cells and memory T cells and NK cells, thus promoting the tumor immunosurveillance effect.^146^ The researchers found that zinc ions released from ZnS@BSA nanoclusters under acidic TIM significantly enhanced cGAS/STING signaling and promoted the function of CD8+ T cells and DCs, which improved the immunotherapy effect of HCC^147^ (Figure 3).

Immunogenic cell death (ICD) is a novel tumor cell death manner that could promote the antitumor immune responses. Recent studies have indicated that some ions may modulate tumor immunity by triggering the ICD. For example, researchers used nanomaterials to construct a cocarrier with Cu^2+^ and DSF, which generate the cytotoxic metabolite DTC‐Copper complex for tumor therapy. One of the active metabolites, CuET, can effectively induce ICD to modulate the TIME.^148^ In addition, other ions such as calcium was also reported to induce ICD in cancer.^149^ Therefore, ion metabolism targeting immune cells is also significant part in cancer immune therapy, and their roles and mechanisms for tumor immunotherapy are worth exploring. With the development of nano‐based therapeutic biomolecule immunotherapies, targeted ion metabolism will have a broader future.

Cancer cells produce metabolites that can have profound effects on immune cells in the TIME. Targeting metabolic pathways in tumors facilitates alteration of metabolic competition between tumors and immune cells that increases the immunogenicity of tumors. The metabolic products produced by tumor cells are various and have different effects on immune cells. Immune cells can employ a variety of programs to respond to different incentives in the microenvironment, and they need to adapt to the cellular metabolism reprogramming in order to match the biochemical demands for each different functional state. For example, different states of T cell activation need metabolic programs appropriate to their functional requirements. Transitions among states are associated with active cellular metabolic reprogramming. And this requires the coordinated adaptation by a network of signaling and transcription factors for each transition.^150^ The shift from a resting to an activated state of immune cells involves the distribution of nutrients into different pathways, and therefore studying how metabolic pathways are modulated to directly change the immune cell function is critical to the immunotherapy of tumors.

## 5 COMMUNICATION AMONG IMMUNE CELLS IN CANCER

The immune system is a highly complex and dynamic network that relies on sophisticated regulation and communication between immune cells to maintain the health of the organism and combat disease. This communication mechanism involves a series of complex signaling pathways, including exosomes, and cytokine release and surface molecule interactions in the TIME. The aim of this section is to explore recent research advances in immune cell communication, focusing on the role of these communication mechanisms in cancer immunity.

### 5.1 Exosome

Exosomes, as an emerging part of tumor–host interactions, which are key mediators of signal transfer between secretory cells and target cells, are now increasingly considered as message carriers and key molecules in TIME. Exosomes derived from immune cells have immunomodulatory properties and promising therapeutic properties that providing an important role in cancer therapy. For example, M2 macrophage‐derived exosomes miR21 interacts with CD8+ T cells through inhibiting the expression of PEG3, which results in a decrease amount of CD8+T cells and inhibits immune response in glioma.^151^ In addition, M1 macrophage‐derived exosomes can facilitate T‐cell production of IFN‐γ and thus promoting immune response.^152^ DC‐derived exosomes directly interact with specific cytotoxic T lymphocytes (CTL) and promote the activation of CD8+ and CD4+ T cells to inhibit tumor growth.^153^ DC‐derived exosomes express IL‐15Rα, leading to amplification of NKs and secretion of IFN‐γ, which induces tumor regression.^154^ B cell‐derived exosomes with CD73, CD19, CD39, C3, FasL proteins, and integrins on their surface can regulate T cells function and thus influence tumor progression through some specific factors.^155^ MHCII(+) FasL(+) exosomes secreted by B cells that induce the apoptosis of CD4+ T cell.^156^ Treg‐derived exosomes containing miR‐150‐5p and miR‐142‐3p can be delivered to DCs to produce more IL‐10 and less IL‐6 thereby suppressing immune cell activity.^157^ TAM‐secreted exosomes containing miR‐21‐5p and miR‐29‐3p promote the progression of epithelial ovarian cancer by suppressing STAT3 expression and inducing an imbalance of Treg/T helper cell (Th)17.^158^ In addition, mast cell‐derived exosomes contained heat shock proteins have been reported to induce DC maturation.^159^ NK cells secrete exosomes contained miR‐186 that stimulate other immune cell (such as T cells and monocytes) functions and also attenuate the immunosuppressive effects of tumor cells by reducing PD1 expression.^160^ As mentioned above, a variety of immune cells possess the function of secreting exosomes, which contain complex biological signals that interact with other immune cells as well as tumor cells, thereby killing tumor cells through a variety of mechanisms. Exosomes carry biomolecules with the capacity to regulate the immune response, such as cytokines, miRNAs, and other regulatory substances. These molecules, delivered through exosomes, can influence the immune status of recipient cells and thus regulate the activity and function of immune cells. Exosomes transmit information between different types of immune cells, prompting them to collaborate with each other to perform specific immune tasks. This intercellular communication helps to create synergies and increase the efficiency of the immune response.

### 5.2 TIME

The TIME, which offers conditions for the tumor cells to grow, is a deeply immunosuppressive environment. In this section, we will focus on immune cell types that are directly engaged in regulating tumor‐killing activity, such as T cells, NK cells, and MDSC. Among them, MDSC are considered to be the immune cells that make the greatest contribution to the development of the TIME.^161^ The MDSC usually including tumor‐associated macrophages (TAM) and tumor‐associated neutrophils. MDSC can not only act as a source of TAM in tumors, it can also influence macrophage polarization, activation and function.^162^ In the tumor environment, MDSC produce IL10 to regulate IL12 production in macrophages, thus playing an important role in macrophages’ polarization toward the M2 phenotype.^163^ In addition, MDSC have also been reported to affect MHC II expression in macrophages possibly.^164^ The interactions that occur between MDSC and macrophages aggravate the suppression of immune cells in TIME through changing the production of cytokines and the expression of some key molecules. However, there is very few literatures to introduce the interaction between neutrophil and MDSC, and these field remains to be investigated.

As reported, researchers cocultured MDSCs with NK cells and found that the MDSCs can inhibit the tumor cytotoxic activity of NK cells by regulating TGFβ and resulted in immune tolerance.^165^ Previous studies have suggested that the ability of MDSC to mediate NK cell unresponsiveness may be related to the MDSCs‐mediated downregulation of CD247 expression on the surface of NK cells.^166^ Furthermore, MDSCs can also excrete diverse soluble elements, such as nitric oxide‐inducible nitrogen oxygenase and ROS in order to enhance immunosuppression and suppress NK cell activation.^167^ In addition, MDSCs also generate IDO to reduce the expression of DNAM1, NKG2D, and NCR, thereby blocking NK cell activation.^168^

Recently, it was demonstrated that MDSCs could secrete itaconate, which could be absorbed by CD8+ T cells and inhibit the properties of CD8+ T cells proliferation and also inhibit the production and activity of cytokine, thus promoting the growth of melanoma.^169^ Another study demonstrated that dysregulation of notch signaling in immature T cells facilitated the formation of CD11b+Gr‐1+ MDSCs, while deficiency of anti‐Gr‐1‐mediated MDSCs reduces the malignant T‐cell proliferation and expansion in acute lymphoblastic leukemia (ALL).^170^ In oral squamous cell carcinoma, MDSCs induced the generation of Treg cells by inhibiting TGF‐β and promoted the differentiation of Th17 cells by secreting PGE2, IL‐1β, IL‐6, and IL‐23.^171^ In addition, MDSC facilitate natural Treg cell expansion and promote the development of induced Treg cells by producing IFN‐γ, TGFβ, and IL‐10.^172^ MDSC suppresses T cell activation by generating ROS and RNS and depleting arginine and cysteine, which are both required for the activation and proliferation of T cells.^173^ In addition, mutual crosstalk between T follicular helper cells and B cells in TLSs plays an important function in antitumor immunotherapy.^174^

MDSC can increase immunosuppressive function through interactions with DC. The impaired cross‐presentation of DC in cancer may be one of the barriers to successful cancer immunotherapy. In melanoma, MDSC reduces antigen uptake and prevents DC maturation, while simultaneously obstructing the capacity of DCs to activate T cells.^175^ It has been reported that MDSC prevents cross‐presentation of DC while not interfering the direct presentation of DC to antigen, which process was linked to lipid peroxidation and requires no direct cell‐to‐cell contact.^176^ The MDSCs have been shown to inhibit not only DC function, but also their maturation and progression. A recent study examined the expression of CD11b and CD33 (MDSC markers) as well as CD303 and IDO1 (DC markers) in patients with OSCC and analyzed their relationship with clinicopathological parameters, and the results showed a positive correlation between these biomarkers. In addition, MDSC and DC were highly expressed in OSCC patients, suggesting that MDSCs and DCs infiltration was significantly correlated with the progression of OSCC.^177^

**FIGURE 4:**
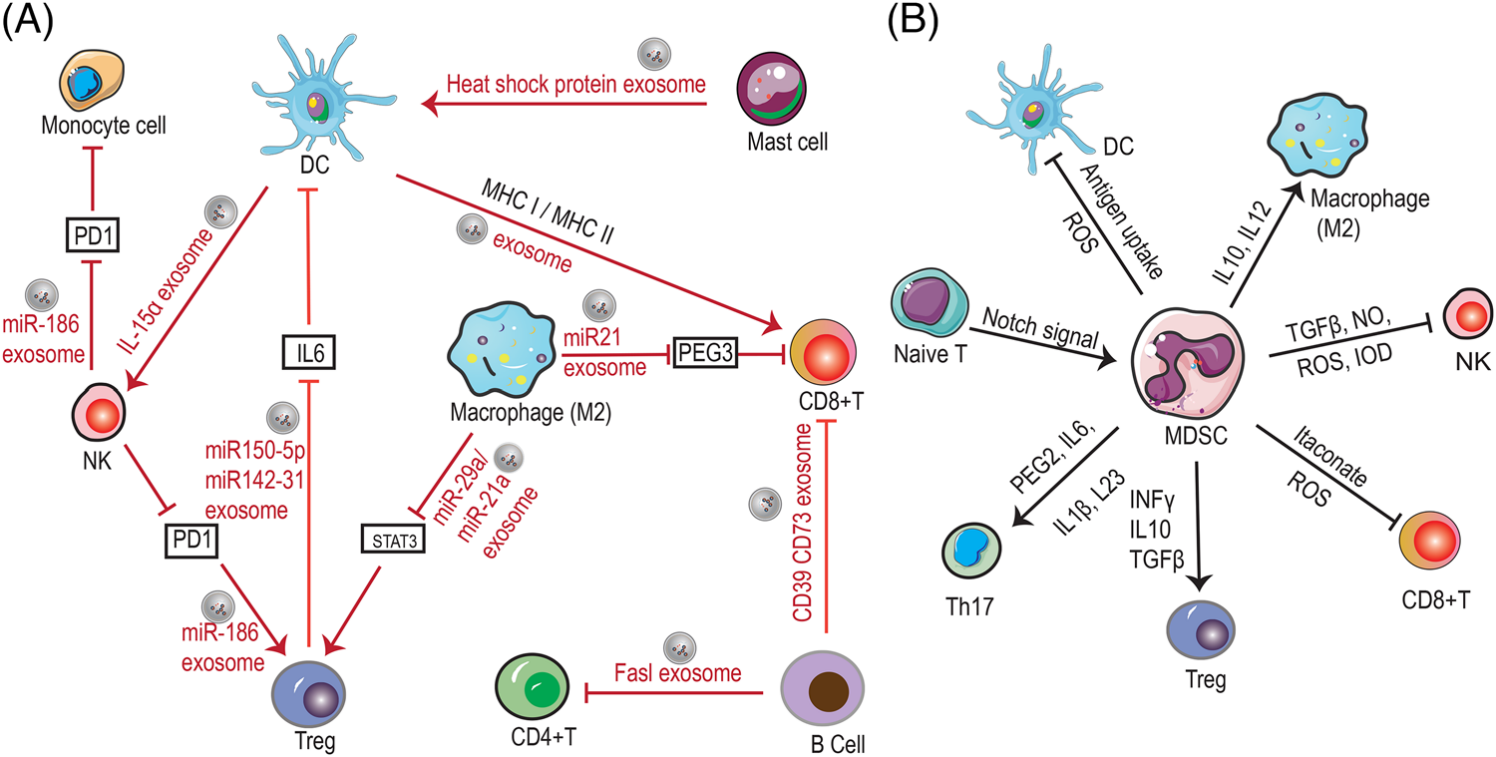
Communication among immune cells. (A) Immune cells communicate with each other by secreting exosomes. Exosomes can carry biomolecules with the capacity to regulate the immune response, such as cytokines, miRNAs, and other regulatory substances. These molecules, delivered through exosomes, can influence the immune status of recipient cells and thus regulate the activity and function of immune cells. (B) Immune cells can utilize components of the TIME for cell communication. MDSCs are the immune cells that contribute most to the development of the tumor immunosuppressive microenvironment. In TIME, MDSCs create an immunosuppressive environment that facilitates tumor escape and progression through a variety of mechanisms, including inhibition of T cells, NK cells, DC cells, triggering of Tregs and M2 macrophages. The red arrows in the figure indicate that immune cells communicate with each other by secreting exosomes, and the black arrows indicate that they communicate through TIME components. (MDSC, myeloid‐derived suppressor cells).

In addition, apart from immune cells, TIME contains fibroblasts, endothelial cells, epithelial cells, and so on; these nonhematopoietic cells have the ability to generate immunomodulatory factors and also participate in the interactions between the tumor and the immune system.^178^ Some components of TIME can produce cytokines, chemokines, and so on, which modulate the function of immune cells through a variety of signaling mechanisms. For instance, cancer‐associated fibroblasts modulates the tumor immune response by secreting cytokines, chemokines, and so on, which regulate macrophage polarization^179^, ^180^ and influence the interaction between immune cells such as DC cells and T cells.^181^, ^182^ In addition, TIME also have affected the activity of immune cells and thus impacted their ability to attack cancer cells. Comprehension of the interactions between immune cells in TIME is crucial for the clinical application of tumor immunotherapy (Figure 4).

The communications between immune cells have important implications in cancer immunotherapy. In recent years, some bispecific antibodies have brought different immune cells closer together (e.g., DCs and T cells) and increased mutual communication, which is considered a novel cancer immunotherapy option.^183^, ^184^ This novel therapy using bispecific antibodies to increase DC‐T cell interactions promotes both T cell activation and function as well as cDC1 maturation.^185^ On the one hand, communication between immune cells helps modulate and coordinate the immune response. Different types of immune cells can interact with each other through cytokines, chemokines, and other signaling molecules to ensure the timely, intensity, and direction of the immune response. On the other hand, lack of communication or miscommunication between immune cells might result in the inability of immune cells to recognize and attack tumor cells in a prompt manner. Thus, tumor cells could take advantage of the communication between immune cells to establish immune tolerance and thus evade immune attack. In conclusion, understanding and regulating the communication between different immune cells can provide important guidance for the development of more effective immunotherapies.

## 6 RELATIONSHIPS BETWEEN IMMUNE CELL METABOLISM, EPIGENETICS MODULATION, AND CANCER IMMUNOTHERAPY CLINICAL EFFICIENCY

Available evidence demonstrates that metabolism and epigenetics can collectively regulate immunotherapy efficiency. The metabolic modifications, epigenetic remodeling, and transcriptional modulation influenced the reactivation of exhausted CD8+ T cells in the TIME, which are critical regulators for cancer immunotherapy.^186^ Immune cells require large amounts of energy and metabolites to maintain their activity in response to tumors. And the immune cell response to cancer is largely relying on the immune cell type and functionally specific metabolic programs. So, an intervention in metabolism can significantly improve the efficacy of cancer immunotherapy.^187^ A variety of metabolic mechanisms can coordinate with immune cell behaviors in response to reprogramming in the TIME, which modulates the efficacy of antitumor immunity.^188^ NF‐κB‐inducible kinase promotes CD8+ T cell metabolism and activation through metabolic reprogramming toward aerobic glycolysis, thereby enhancing the therapeutic efficacy of T cell adoptive therapy (tumor size reduction).^189^ Studies have shown that reduction of glutamine activates CD8 function, leading to better clinical benefits of neoadjuvant immunotherapies in combination with ICI therapy.^190^ Nicotinamide phosphoribosyltransferase (NAMPT) was necessary for the activation of T cells, and supplementation with NAD(+) could augment the tumor‐killing effect of T cells in CAR‐T and ICI treatment by rescuing TUB‐mediated NAMPT transcription in T cells.^191^ In addition, monoamine oxidase A, an important enzyme in the metabolism of monoamines, could lead to T cell dysfunction, which reduces the cancer immunotherapy efficacy of immune checkpoint therapy and is strongly associated with lower survival of various cancers.^192^ In addition, targeting the metabolism of MDSC contributes to the efficacy of antitumor immunotherapy.^193^ Tumor‐derived immunoglobulin‐like transcript 4 (ILT4) and PIR‐B (ILT4 homolog) induce effector T cell senescence by activating the MAPK ERK1/2 signaling pathway to increase fatty acid synthesis and lipid accumulation in tumor cells, increased the survival in patients with ICI treatment.^194^ The researchers discovered that the tumor‐infiltrating T cells (especially CD8 T cell exhaustion and Treg suppression) presented the highest active levels of Gln metabolism among immune cells by building a quantitative system of Gln metabolism, which can be used to predict the prognosis and the immunotherapy efficacy including ICI and T cell adoptive therapy in lung cancer.^195^

The epigenetic gene PR domain zinc finger protein 1 (PRDM1) knockdown in T cells showed an increased chromatin accessibility and promoted the expansion of memory CAR‐T cells, which enhanced T cell durability and improved the efficacy of cancer immunotherapy and improved survival rate.^196^ The ubiquitin ligase MDM2 reduces c‐Cbl‐mediated STAT5 degradation by competitive binding to STAT5 with c‐Cbl, thereby enhancing tumor‐infiltrating CD8+ T cell‐mediated antitumor immunotherapy (tumor size reduction).^197^ Studies have shown that DNA‐methylated CD8+ TIL cells are reported to assess immune response and prognosis in CRC.^198^ In addition, the m6A modifiers have also been considered as biomarkers for assessing cancer immunotherapy response. Researchers constructed the m6A regulatory prognostic risk score (MRPRS) to assess the survival and immunotherapy efficacy in clear cell renal cell carcinoma, revealing that MRPRS is a potential hopeful biomarker for immunotherapy response assessment.^199^ Protein tyrosine phosphatase receptor type O significantly affects CD8+ T cell infiltration in breast cancer and it could be used to predict immunotherapy efficacy and prognosis in breast cancer.^200^ Furthermore, the epigenetic reader SP140, a regulator of the macrophage transcriptional program, was found to trigger IFN‐γ production in TAM by inhibiting STAT1 transcription and phosphorylation. And higher level of SP140 was associated with higher immune scores, infiltration of CD8+ T cells and TAM, which was a potential predictive biomarker of immunotherapeutic response in metastatic melanoma.^201^

**FIGURE 5:**
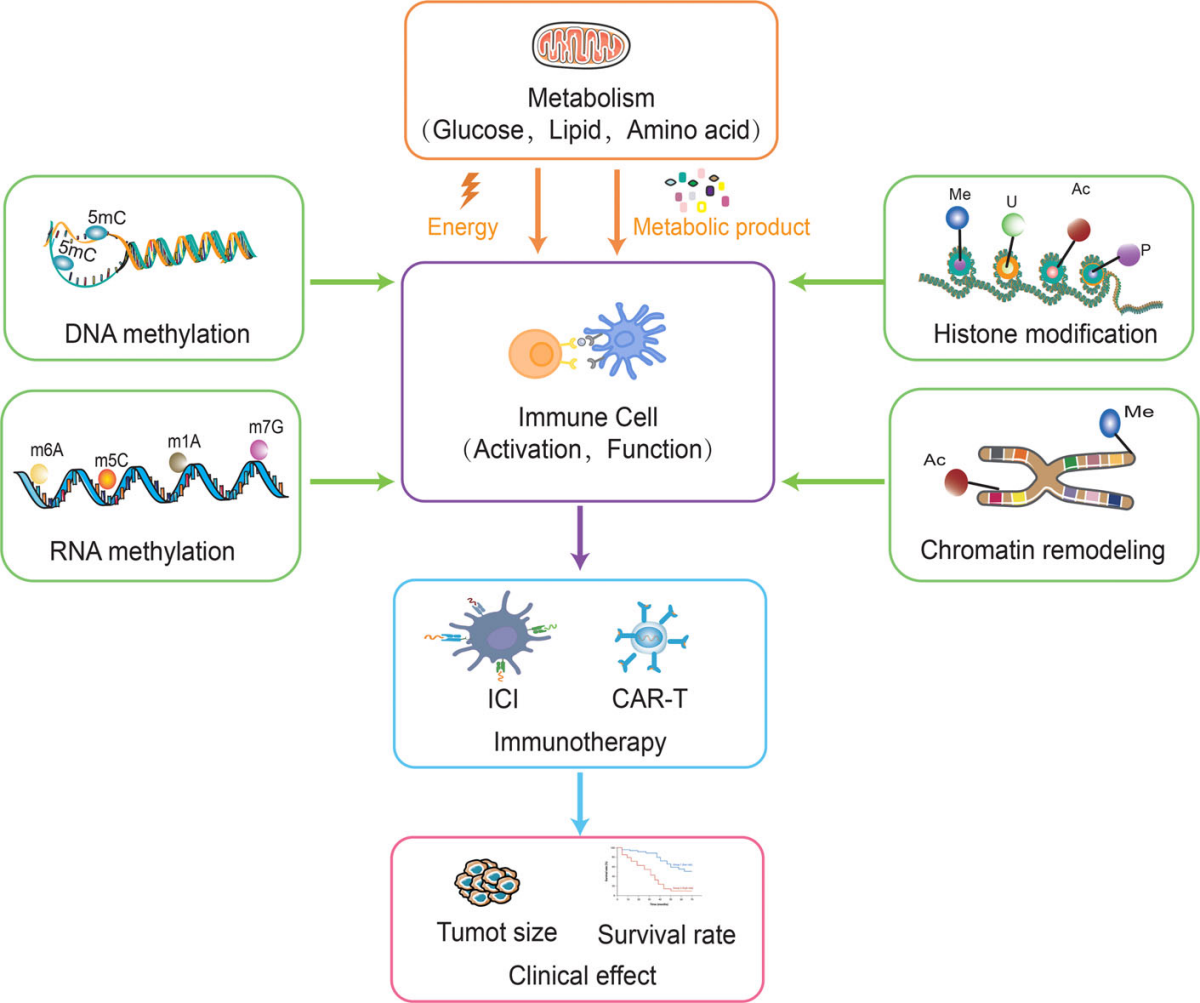
Relationships between immune cell metabolism, epigenetics modulation and cancer immunotherapy clinical efficiency. Epigenetic regulation (including DNA methylation, RNA methylation, histone modification and chromatin remodeling) of immune cells can influence the differentiation and activity of immune cells, while metabolic status influences the energy supply and reactivity of immune cells, and they are participated in cancer immunotherapy and influenced clinical effect through immune cells as a bridge. (Me, methylation; U, ubiquitination; Ac, acetylation; P, phosphorylation).

Metabolic reprogramming and epigenetic reprogramming are connected to each other, and to a large extent, metabolic status determines the epigenetics of cancer. Epigenetics modulates the expression of metabolism‐related genes and thus exerts an essential role in the metabolism of cancer cells. Epigenetic modifications can also affect the transcriptional regulation of immune cells, thereby influencing the activation and inhibition of their metabolic pathways. Meanwhile, metabolic fluxes are engaged in epigenetic modulation through impact on biosynthesis and energy production of macromolecules such as nucleic acids and proteins. Epigenetic regulation of immune cells can influence the differentiation and activity of immune cells, while metabolic status influences the energy supply and reactivity of immune cells, and they are participated in cancer immunotherapy through immune cells as a bridge (Figure 5). Therefore, exploring the interplay between epigenetic and metabolic regulation in immune cells could provide new ideas for cancer immunotherapy.

## 7 CANCER IMMUNOTHERAPY: INHIBITOR APPLICATIONS, CLINICAL TRIALS, BIOMARKERS, AND MOUSE MODELS

### 7.1 Inhibitors in the field of cancer immunotherapy

Different immune cell compositions in TIME can influence the immune checkpoint inhibitor (ICI) response to tumor therapy.^202^, ^203^ Immune checkpoint receptors such as CTL‐associated protein 4 (CTLA4), PD1, T‐cell immunoreceptor with Ig and ITIM structural domains (TIGIT), and lymphocyte activation gene 3 (LAG3) could modulate the activation, differentiation, and function of T cells. For example, PD1 directly modulates TCR signaling to attenuate T cell activity. Additionally, deficiency of CTLA4 in Tregs induces aberrant T cell activation and causes autoimmunity. And deletion of Treg could counteract the expansion of Treg cells induced by CTLA4 blockade, thereby improving the efficacy of anti‐CTLA4 therapy.^204^ Anti‐TIGIT targeting Tregs possesses strong potential for cancer immunotherapy,^205^ and targeting Tregs may also contribute to cancer immunotherapy efficacy when used as monotherapy or in combination with ICB antibodies.^206^ Recent studies have shown that sialic acid‐binding immunoglobulin‐like lectin (Siglec) receptors on TIICs have an interplay with glycan‐containing sialic acid in the tumor microenvironment, which could be a novel immune checkpoint and a promising potential target for cancer immunotherapy. The Siglec receptor is widely expressed on different immune cells including T cells, DCs, NK cells, neutrophils, and macrophages. And the interaction between Siglec receptors and sialic acid glycan ligands on these immune cells helps to establish an immune suppressive microenvironment and suppress antitumor immunity in the cancer microenvironment.^207^ More importantly, studies have reported that Siglec‐15 can be targeted by the blocking antibody NC318, and it has been shown in early clinical trials that the antibody is responsive to cancer immunotherapy in some patients.^208^ This suggests that various immune checkpoints can influence tumor immunotherapy by influencing the function and activation of immune cells and modulating the activity and specificity of the immune system.

ICI therapy is one of the most widely utilized cancer immunotherapies at present. Currently, drugs against immune checkpoints focus on targets such as PD1, PDL1, and CTLA4. Among them, PD1 inhibitor nivolumab (trade name Opdivo) used to treat a variety of cancers, including melanoma, NSCLC, renal cell carcinoma, and head and neck cancer.^209^, ^210^, ^211^, ^212^ Pembrolizumab (Keytruda): used to treat melanoma, NSCLC, head and neck cancer, Hodgkin's lymphoma, and so on.^213^, ^214^, ^215^ And PDL1 inhibitor, atezolizumab (Tecentriq), is mainly used for the treatment of uroepithelial cancer, HCC, and NSCLC.^216^, ^217^, ^218^ Avelumab (trade name Bavencio) is mainly used for the treatment of Merkel cell carcinoma and uroepithelial carcinoma.^219^, ^220^ Durvalumab (trade name Imfinzi) is mainly used for the treatment of NSCLC and bladder cancer.^221^ Moreover, CTLA4 Inhibitors Ipilimumab (trade name Yervoy) initially used to treat advanced melanoma, now also used for other cancer types.^222^ Notably, the range of indications for these drugs will continue to expand based on new clinical trial results and regulatory approvals. In addition, certain drugs may be more effective in specific patient populations.

While ICIs have achieved remarkable success in cancer treatment, they may also affect noncancer cells, leading to a range of immune‐related side effects such as autoimmune reactions, inflammatory responses, and so on.^223^, ^224^ Some patients may acquire resistance to ICIs, and further research is needed to address the challenges associated with side effect management, resistance, treatment failure, and cost and accessibility. The combination of ICIs with other treatments, such as radiotherapy, chemotherapy, targeted therapy, or other immunotherapies, is being studied extensively. Such combination therapies may improve treatment efficacy and overcome the limitations of monotherapy.

### 7.2 Latest clinical trials of immune cell‐based cancer immunotherapy

There are two main types of immune cell‐based cancer immunotherapies currently on the market or approved, namely DC therapy and CAR‐T cell therapy. In 2010, the DC therapy Sipuleucel‐T (trade name: Provenge) was approved for the treatment of metastatic desmoplasia‐resistant prostate cancer that is asymptomatic or only mildly symptomatic.^225^, ^226^ In 2017, the CAR‐T therapy Tisagenlecleucel (trade name: Kymriah) was approved for the treatment of ALL,^227^, ^228^ and in the same year, the Axicabtagene ciloleucel (trade name: Yescarta) was approved for the treatment of diffuse large B‐cell lymphoma.^229^, ^230^ In addition, Brexucabtagene Autoleucel (Tecartus) is approved for the treatment of relapsed or refractory B‐cell precursor ALL in adults.^231^ Lisocabtagene Maraleucel (Breyanzi) is approved for the treatment of relapsed or refractory large B‐cell lymphoma.^232^ Additionally, there are also many immune cell therapies in clinical trials.

Clinical trials allow doctors to assess the accuracy and reliability of new diagnostic and therapeutic techniques to provide more appropriate treatment options for patients. Today, immune cell therapies are attracting a lot of attention in cancer immunotherapy, and scientists are continuing to improve therapies and trial methods. Below are some examples of clinical trial results for cancer immunotherapy based on immune cell therapies that have been published in the recent 2 year, providing new discoveries and understanding for fighting cancer (Table 1).

**TABLE 1:** Cancer immunotherapy clinical trials based on immune cell in latest years.

ClinicalTrials number	Cancer type	Patient amounts	Target	Agent	End points	Phase	Recruitment status	References
NCT03607539	NSCLC	397	TIL/PD1	Sintilimab	PFS/OS/ORR	Phase 3	Completed	^233^
NCT02278887	Melanoma	168	TIL/CTLA4	Ipilimumab	PFS/OS	Phase 3	Active, not recruiting	^234^
NCT02652455	Metastatic melanoma	11	TIL/PD1	Nivolumab	Safety/feasibility	Phase 1	Completed	^235^
NCT02421640	Nasopharyngeal carcinoma	156	TIL	–	Safety/objectivity	Phase 1	Unknown states	^236^
NCT04555551	Multiple myeloma	17	CAR‐T/ GPRC5D	lentiviral vector containing GPRC5D CAR	Safety	Phase 1	Active, not recruiting	^237^
NCT04093596	Multiple myeloma	43	CAR‐T/ BCMA	ALLO‐715	Safety/tolerability	Phase 1	Active, not recruiting	^238^
NCT02640209	Chronic lymphocytic leukemia	20	CAR‐T/ CD19/Bruton's tyrosine kinase (BTK)	Ibrutinib	Safety/feasibility	Phase 2	Terminated	^239^
NCT04148430	Lymphoma	31	CAR‐T/ CD19/IL‐1R	Anakinra	Severe (grade≥3) ICANS	Phase 2	Active, not recruiting	^240^

Researchers conducted a large‐scale randomized controlled phase 3 clinical trial (NCT03607539) to explore the relationship between TIL and the efficacy of immunotherapy in 397 advanced NSCLC patients, where the primary endpoint was progression‐free survival (PFS) while OS and objective remission rates (ORR) were secondary endpoints. The results showed that combination therapy of chemotherapy plus sintilimab (anti‐PD1) was associated with statistically significant improvements in PFS (HR = 0.12, 95% CI: 0.06–0.25, *p* < 0.001) and OS (HR = 0.27, 95% CI: 0.13–0.55, *p* < 0.001) only for subtype II (PDL1+ and TIL+) compared with chemotherapy, which further illustrates the critical role of immune cells in cancer immunotherapy.^233^ Recently researchers conducted a phase 3 multicenter open‐label clinical trial (NCT02278887) in which 168 patients with advanced melanoma were randomly assigned to receive either TIL or ipilimumab, with the results revealing a median PFS of 7.2 months (95% CI, 4.2–13.1) and median overall survival was 25.8 months (95% CI, 18.2 to not reached) in the TIL group while PFS of 3.1 months (95% CI, 3.0–4.3) and 18.9 months (95% CI, 13.8–32.6) in ipilimumab group.^234^ Researchers reported a phase I clinical trial (NCT02652455) of nivolumab in combination with TIL ACT for the treatment of melanoma which proved to be a safe and feasible treatment.^235^ A phase I clinical trial (NCT02421640) of 156 nasopharyngeal carcinoma patients using TILs following concurrent chemoradiotherapy was determined to be safe and objective.^236^ This demonstrates the potential survival benefit of TILs in patients with low levels of circulating CD8+ T cells and PDL1 expression.

Recent results from a Phase I clinical trial (NCT04555551) of CAR‐T cell therapy (MCARH109) targeting GPRC5D confirmed that GPRC5D is an important immunotherapeutic target in multiple myeloma.^237^ Researchers conducted a phase 1 clinical trial (NCT04093596) in 43 patients with multiple myeloma evaluating allogeneic anti‐BCMA CAR‐T cell therapy (ALLO‐715). Overall, 24 patients (55.8%) responded, with 17 (70.8%) in remission, 11 (45.8%) in partial remission or better, and six (25%) in complete remission, demonstrating the viability and safety of allogeneic CAR‐T cell therapy.^238^ In addition, a phase II clinical trial (NCT02640209) of autologous anti‐CD19 humanized binding domain T cells (huCART‐19) added to Bruton's tyrosine kinase (BTK) inhibitor ibrutinib for 20 patients with B‐cell chronic lymphatic leukemia and the results indicated that patients in the huCART‐19 group had higher rates of remission with favorable safety and feasibility outcomes.^239^ Investigators conducted a phase II clinical trial (NCT04148430) of the IL‐1 receptor antagonist (IL‐1Ra) anakinra in patients with relapsed/refractory large B‐cell lymphoma who were treated with anti‐CD19 CAR‐T cells. The results showed that anakinra treatment reduced immune effector cell‐associated neurotoxicity syndrome (ICANS) while not damaging the efficacy of anti‐CD19 CAR‐T therapy.^240^

In addition, there are many ongoing clinical trials of immune cell‐based cancer immunotherapies, including NKT cell therapy, NK cell therapy, CAR‐M, LAK cell therapy, TCR‐T cell therapy, DC‐CIK cell therapy, and so on. For details on the progress of these clinical trials, please refer to https://clinicaltrials.gov/. Among them, CAR‐M and NK cell therapies are already in the late clinical stages, and the results of their clinical trials are extremely promising.^241^

### 7.3 Potentially available biomarkers for evaluation of cancer immunotherapy

There is no doubt that the advent of immunotherapy has provided a new and more effective therapy for the prevention and treatment of tumors. However, it is urgent to evaluate and predict the efficacy of immunotherapy and thus improving the effectiveness of immunotherapy, since immunotherapy cannot be suitable for all patients. The Cancer ImmunoMonitoring and Analysis Center and the Cancer ImmunoData Commons have been established and are able to systematically identify biomarkers and correlate them with clinical outcomes, which is essential for enhancing immunotherapeutic efficiency for cancer patients.^242^

It has been believed that CTLA4, TMB, and SMACA4 mutations are considered as potential predictive biomarkers of ICB efficacy in EBV virus‐associated gastric cancer.^243^ A recent study showed that the expression of PDL1, B7‐H3 and VISTA as well as some tumor necrosis factor receptor superfamily such as OX40L, CD27, 4‐1BB, CD40, and CD95/Fas were correlated with the head and neck squamous cell carcinoma immunotherapy efficiency, and could serve as potential biomarkers to predict the immunotherapy response.^244^ Using meta‐analysis, Zhou et al.^245^ discovered that high expression of PDL1 in tumor cells instead of tumor tissues was associated with better prognosis, and PDL1 could be used as a prognostic biomarker of immunotherapy efficacy for HCC.

However, tumor tissue biopsies are invasive and cannot obtain the full spatial heterogeneity of the tumor, as well as being difficult to follow up on treatment efficacy. In recent years, it has been found that cancer immunotherapy efficacy can be reflected by comprehensive analysis of systemic immune events through peripheral blood immune cell subpopulations. Thus, circular immune cell‐based biomarkers can assess and provide guidance for cancer immunotherapy.^246^ Liquid biopsies can be used to assess the progression of tumors and the efficacy of clinical therapies during cancer immunotherapy. Among them, ctDNA could be used as a tumor load biomarker to assess the efficacy of immunotherapies.^247^ In addition, PDL1 in circulating tumor cells and CD4/CD8+ T cell populations may serve as prognostic biomarkers for immunotherapy in metastatic genitourinary cancer patients.^248^ GNG4 is expressed typically in exhausted CD4+ T cells, and its high expression is correlated with a higher immune cell infiltration level and a better immunotherapy response, which could be recognized as a biomarker for assessing the immunotherapy efficacy in bladder cancer.^249^ A recent study has indicated that the tumor mutational load in blood is expected to be a biomarker for the prediction of the efficacy of immunotherapy in NSCLC.^250^ Researchers found that patients with high expression of LAG‐3 in the surface of CD8+ T cells in peripheral blood were less responsive to immunotherapy through analysis of peripheral blood cells from melanoma patients. At the same time, they suggested that LAG‐3 protein could serve as a potentially vital biomarker for predicting the efficacy of ICB.^251^ Study demonstrates that circulating extracellular vesicles expressing CD81 may serve as a possible biomarker of ICI response in patients with low levels of PDL1 expression in advanced NSCLC.^252^ Serum biomarkers, the CEA and Ca‐125, potentially serve as biomarkers for predicting the immunotherapy efficacy in NSCLC treated with PDL1 inhibitors.^253^ Circulating soluble programmed death ligand 1 (sPDL1) is a prospective dynamic biomarker that negatively regulates the function of T cells, evaluates the efficacy of immunotherapy in pMMR CRCs, and can serve as a predictive prognostic marker in a diverse range of cancers.^254^

In recent years, with the development of bioinformatics, some novel potential markers to predict cancer immunotherapy efficacy have been identified such as PTTG,^255^ EXTL3,^256^ CTNNB1,^257^ CXCL5,^258^ APC,^259^ and TASL.^260^ Researchers identified 11 immune‐related gene prognostic indicators that have the capacity to predict immune cell infiltration in the tumor microenvironment, the efficacy of immunotherapy and can more accurately predict the survival of HCC patients.^261^ In addition, the researchers compiled a list of 52 candidate genes that could possibly predict the efficacy of ICB therapy, based on data from a cohort of 350 patients with NSCLC. These candidate genes are characterized by compound mutations that might be better predictors than TMB and could serve as new biomarkers for assessing the NSCLC immunotherapy efficacy.^262^ Researchers developed a computational model of resilient T cells (Tres) using single‐cell transcriptomics data to recognize immunosuppressive signatures (e.g., TGFβ1 and PGE2). It is a reliable predictor of clinical response to immunotherapy in a wide range of tumors including melanoma, TNBC, and lung cancer. The Tres revealed that FIBP is the major negative biomarker of tumor resilient T cells and can be used to assess the immunotherapy response in solid tumors.^263^ An immune infiltration score (IIS) was calculated based on immune cell infiltration in the cancer microenvironment. And patients with higher IIS had significantly higher PDL1 expression and shorter PFS time, which can be used as a novel and reliable biomarker to assess the prognosis of TNBC patients.^264^ A recent study found that using spatial quantification of CD8 and PDL1 markers to quantify immune scoring is a novel in vitro diagnostic methods, which is capable to predict the efficacy of anti‐PD1/PDL1 immunotherapy in NSCLC, and is a potential powerful tool for evaluating the outcomes of cancer immunotherapy.^265^ The researchers tried a new method to automatically select tissues and calculate the frequency of cell to cell spatial interactions happening in the PD1/PDL1 pathway, which could be used to reveal spatial interactions for immune escape and provide potential prognostic information for oropharyngeal squamous cell carcinoma.^266^

In addition to the classical immune checkpoints and immune cells, other new areas such as cancer stemness, ferroptosis, pyroptosis, lncRNAs, and radiogenomic traits have also been considered as potential markers for cancer immunotherapy efficiency. Researchers have found that cancer stemness is a key reason for ICI resistance by comprehensively analyzing single‐cell and bulk RNA sequencing data, and that cancer stemness gene expression profiles can be used as biomarkers of cancer immunotherapy efficacy.^267^ In addition, the ferroptosis score is strongly associated with the efficacy of immunotherapy in colon cancer. Where patients with high ferroptosis score (characterized by high TMB and MSI‐high subtype) showed poor prognosis using immunotherapy. Among them, ALOX5 is a key ferroptosis gene identified as a predictive marker for immunotherapy efficacy.^268^ Yet another study found that cancer patients with high ferroptosis scores displayed high CD8+ T cell and TIL infiltration as well as enrichment of immune‐related signaling pathways, which could be a good prognostic factor and an assessment of the immunotherapy effect.^269^ In addition, the researchers also elucidated that tumor‐infiltrating B‐lymphocyte lncRNA signature can be used as an index of immune cell infiltration in TIME and lncRNA is expected to be used as a potential biomarker for the prediction of immunotherapy response in bladder cancer.^270^ Also, some pyroptosis‐related genes and immune‐related lncRNAs have been recognized as potential prognostic markers of cancer immunotherapy.^271^, ^272^ In addition, radiogenomic biomarkers to predict therapeutic response to PD1/PDL1 immunotherapy in NSCLC have also been discovered.^273^

**FIGURE 6:**
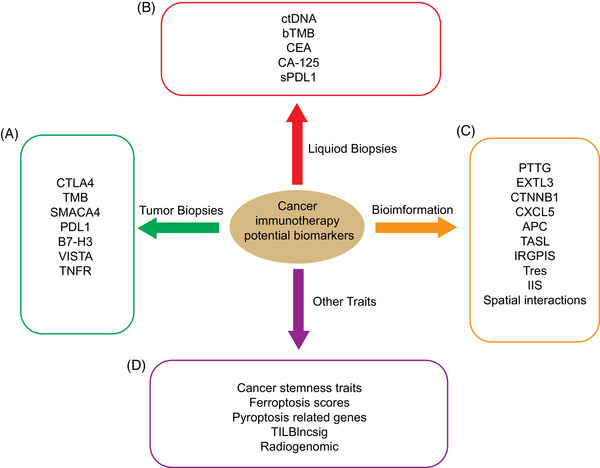
Potentially available biomarkers for evaluation of cancer immunotherapy. (A) Potentially available biomarkers for tumor tissue biopsies. (B) Potentially available biomarkers for liquid biopsies. (C) Potentially available biomarkers identified through bioinformatics methods. (D) Potentially available biomarkers discovered based on other emerging features.

In summary, cancer immunotherapy is a rapidly evolving field, and a growing number of potentially usable biomarkers for predicting and evaluating cancer immunotherapy have emerged. Taken together, these biomarkers allow researchers and clinicians to better understand which patients are most likely to benefit from immunotherapy, thereby personalizing treatment plans and improving outcomes. Currently, there are two major limitations in evaluating potential biomarkers for cancer immunotherapy: first, although many promising markers have been identified, such as PD‐L1 expression, tumor mutational load (TMB), and immune cell infiltration, the predictive power of these markers is not always consistent and exhibits variability in different types of cancer. Second, the process of biomarker discovery and validation is complex and time consuming, and there is a lack of uniform standards and methods to assess and validate the clinical application value of new markers, which limits their widespread use in personalized immunotherapy. As more studies are conducted, it is expected that additional biomarkers will be identified and existing assessment methods will be improved (Figure 6).

### 7.4 Mouse tumor models for immunotherapy

Immunotherapy has yielded benefits for some patients with tumors. However, cure rates for most patients remain low, and many patients do not even respond to these treatments. Preclinical in vivo models play a crucial role in the clinical efficacy assessment of tumor immunotherapy. Mouse tumor models are one of the key tools for studying tumor pathogenesis. In recent years, with the advances of tumor immunotherapy, mouse models have been further developed.^274^ Currently, the main mouse models for tumor immune efficacy evaluation include syngeneic model, genetically engineered mouse models, humanized mouse models with immune system reconstitution.

#### 7.4.1 Syngeneic mouse model

Syngeneic tumor models are the most commonly used preclinical models, and these models are particularly important in the evaluation of immuno‐oncology, which have an intact immune system and can be used to investigate the process of antitumor immune response. A recent study performed tumor immunoassays through two syngeneic mouse model (CT‐26 and Colon 26), which have similar genetic backgrounds with different sensitivities to anti‐PD1 therapy, revealing the underlying mechanisms of anti‐PD1 responses. And the results suggest that Wnt pathway is associated with anti‐PD1 responses and may be the major factor differentiating these two models.^275^ A subset of syngeneic resistance models can be used as an analog of the resistance heterogeneity encountered in the clinic and can be used for mechanism exploration.^276^ Recently, researchers have established a mouse syngeneic model of breast cancer. The model can be successfully used to analyze the efficacy of ICB in combination with predictive biomarkers of breast cancer in immuno‐oncology therapy.^277^ In addition, with a homozygous pancreatic tumor model, the researchers found that combination of treatment with chemotherapy and ICB induced significant delays in tumor regression and regeneration.^278^ A syngeneic mouse model of glioblastoma revealed the effectiveness of combination therapy and confirmed the power of PARP‐targeted alpha therapy in augmenting the potential of PD1 immune checkpoint blockade.^279^ Notably, although the convenience and availability of syngeneic tumor models have made them the most frequently used preclinical models for assessing immunotherapies, these models still have a fatal drawback: they lack the microenvironmental heterogeneity of cancers.

#### 7.4.2 Genetically engineered mouse models

Using genetic engineering techniques, the expression of tumor‐related genes in the mouse genome can be regulated, which could be used to investigate the pathogenesis of diseases. The application of genetically engineered mouse models in tumor immunotherapy is mainly based on the Cre–loxP system. Conditional knockout mice based on the Cre/loxP system use a tissue‐specific promoter to specifically control Cre expression in specific tissues to achieve knockout or modification of target genes in specific tissue cells. The Cre recombinase frequently used in the Cre–loxP system to target immune cells include macrophage/monocyte, DCs, mast cells, and T and B cells.^280^ For example, researchers use Cre/loxP system to discover that the transcriptional coregulator Ess2 controls CD4+ T cell survival through IL‐7 signaling pathways.^281^ With the use of CRISPR/Cas9 and Cre–LoxP knockout methods, the researchers demonstrated that SOX9 inhibited immune cell infiltration and functionally suppressed the infiltration and activity of CD8(+) T cells and NK cells.^282^ Overall, genetically engineered mouse models in general have TIMEs that are linked to human cancers. However, most genetically engineered mouse models have low tumor mutation loads, which makes it difficult to generate strong antitumor immune responses in these models. In recent years, it has been found that combining the genetically engineered mouse model with UV irradiation could increase the mutational burden of the melanoma model, which undoubtedly provides a favorable direction for the development of the genetically engineered mouse model.^283^

#### 7.4.3 Humanized mouse models with immune system reconstitution

Since the mouse immune system does not have exactly the same composition as the human immune system, scientists have constructed humanized mouse models that interact with human immune system cells to assess immunotherapeutic approaches more accurately and realistically. Humanized mice models are immunodeficient mice cotransplanted with human tumors and immune components, which mainly indicates patient‐derived xenografts (PDX) models with immune system reconstitution.

The methods of immune system reconstitution in humanized mice models mainly include three types below.^284^ One of the most convenient ways is to infuse mature human immune cells directly into SCID mice, thereby obtaining the SCID mice that produce human peripheral blood leukocytes.^285^ Another approach is to transplant human CD34+ hspc to immunodeficient mice for generate human SCID regenerative cell mice, which results in a more integrated hematopoietic system that includes compositions such as innate immune cells, adaptive immune cells erythrocytes, and platelets.^286^ The third method, which is the most complete and sophisticated approach to human immune system transplantation, which consists of transplanting CD34+ hspc from human fetal liver and autologous fetal thymus tissue into SCID mice to produce a kind of model known as BLT (bone marrow, liver, thymus).^287^ Notably, most of these immune system reconstitution experimental approaches used mismatched tumors and immune systems, which requires a concern for HLA matching before investigate the immune system–tumor interactions.

Currently, humanized mouse models for immunotherapy mainly include treatment with specific antibodies, small molecule inhibitors, and CAR‐T/NK therapy.^288^, ^289^, ^290^ Recently, a study has shown that mice reconstituted with umbilical cord blood human CD34+ cells were able to induce recession of the response of PDX tumors to anti‐PD‐1 therapy.^291^ In addition, using humanized NOD/Shi‐scid/IL‐2Rnull mice, researchers found that the pattern of immune cell infiltration in a PDX model of non‐small cell lung cancer correlated with cytokines, genetic mutations, stromal content and PDL1 status.^292^ Moreover, PDX models play an important role in immunotherapy of hematology tumors.^293^, ^294^

The PDX model with immune system reconstitution accurately simulates genomic heterogeneity and microenvironmental factors in the development of tumors and is essential for tumor immunotherapy evaluation. However, there is no mouse model that is fully compatible with human biology, although humanized mice have been extensively studied in improving the efficacy of clinical tumor immunotherapy. So, further refinement of mouse models is still needed in the future.

## 8 DISCUSSIONS AND PERSPECTIVES

The response of cancer immunotherapy differs from that of chemotherapeutic agents. In an effort to standardize the evaluation criteria for assessing the efficacy of immunotherapy in clinical studies, the RECIST Working Group released new standards in late 2017. This guideline evaluates the tumor load variations during cancer treatment to provide critical information on disease progression. More importantly, the standard methods of solid tumor measurement and the definition of objective changes in tumor sizes among this guidance will contribute to consistent implementation, explanation, and analysis of immunotherapy trials for researchers and doctors.^295^

Currently, cancer immunotherapy approaches mainly include (1) using cytokines such as IL2 and interferon to activate and promote the immune cell killing ability of the patient; (2) monoclonal antibody therapy such as ibritumomab, nabumomab, pembrolizumab that targeting immune checkpoints; (3) using adoptive cell transfer therapy such as NK cells, DCs, cytokine‐induced killer (CIK) cells, genetically engineered modified T cells; (4) immunizing patients with tumor vaccines made from the patient's own tumor cells, tumor proteins, peptides, and so on to activate the autoimmunity and enhance the antitumor effect; (5) immunotherapy targeting inhibitory cells such as Treg, TAM, and MDSC. And these immunotherapeutic treatments have achieved a certain degree of effectiveness. For instance, CIK cellular immunotherapy provides a pivotal role in clinical trials for the treatment of cancer.^296^ The CIK cell therapy combination with DCs has been relatively successful in lung cancer clinical trials.^297^, ^298^ Combination therapy with CIK cells and PD1 blocking antibodies showed potential for profound durable responses in NSCLC. And those NSCLC subpopulations with PDL1 positivity and high CD8+ TIL counts had better ORR with the combination of PD1 inhibitors and CIK cells.^299^ However, it is worth emphasizing that the response rate to tumor immunotherapy is still not satisfactory, therefore there is an urgent to find the cause, and identify more biomarkers that can indicate the response rate to immunotherapy and thus improve personalized treatment for tumor patients.

From this review, we understood that epigenetic modifications in cancer are involved in the modulation of immune cell activity and function. Epigenetic regulation influences the interactions between cancer cells and immune cells as well as the state of TIME. Epigenetic regulation can be used as an intervention to induce robust antitumor immunity. Combining epigenetic modulators with cancer immunotherapy solutions is a rational strategy to further enhance the efficacy of immunotherapy and has shown promising applications in clinical studies.

Most studies on tumor metabolism have focused on the overall assessment of in vitro tissue culture samples, yet with the emergence of single‐cell sequencing technologies, the impact of tumor metabolism at the single‐cell level cannot be ignored. In addition, whether metabolic changes are responsive or instructive throughout functional changes in immune cells is now still required more detailed researches. And whether the metabolic dysregulation of immune cells in TIME is due to immune cell‐intrinsic programs or to competition with cancer cells for limited nutrients still requires further study.

The current view is that cancer immunotherapy efficacy largely depends on the viability and function of tumor antigen‐specific CD8+ T cells in the immunosuppressive TIME. So, strategies to improve immune efficacy are mainly focused with CD8+ T cells, but the role of other Immune cells such as NK cells with tumor‐killing function as well as Treg and MDSC with tumor‐suppressive function on cancer immune efficacy is also worth attention. In addition, the ineffectiveness of tumor immunotherapy may be attributed in part to the complex effects of tumor immune escape and the microenvironment. Although several literatures have identified potential biomarkers for assessing the efficacy of immunotherapy and have also reported some models for assessing immunotherapy, there is still a demand for a large amount of clinical data to validate them. In addition, the combination of multiple biomarkers to assess immunotherapy efficacy is also well worth investigating. With further understanding of tumors and immune cells, it is to be expected that the immunotherapy field will achieve more breakthroughs in the future, providing a more powerful weapon to overcome the great challenge of cancer.

## AUTHOR CONTRIBUTIONS

D. X. and Y. T. designed the study. S. Q. drafted the manuscript. S. Q., B. X., Q. W., R. Y., J. S., C. H., and S. L. collected the data and conducted the picture processing. D. X., Y. T., and S. L. revised the manuscript. All authors have read and approved the final version of manuscript.

## CONFLICT OF INTEREST STATEMENT

The authors declare that they have no conflict of interest.

## ETHICS STATEMENT

Not applicable.

## Data Availability

All data generated or analyzed during this study are included in this published article.

## References

[1] Advancing cancer therapy. Nat Cancer. 2021;2(3):245‐246. 35121963 10.1038/s43018-021-00192-x

[2] Dagher OK , Schwab RD , Brookens SK . Advances in cancer immunotherapies. Cell. 2023;186(8):1814‐1814. e1811. 37059073 10.1016/j.cell.2023.02.039

[3] Esteve‐Puig R , Bueno‐Costa A , Esteller M . Writers, readers and erasers of RNA modifications in cancer. Cancer Lett. 2020;474:127‐137. 31991154 10.1016/j.canlet.2020.01.021

[4] Nanamori H , Sawada Y . Epigenetic modification of PD‐1/PD‐L1‐mediated cancer immunotherapy against melanoma. Int J Mol Sci. 2022;23(3):1119. 35163049 10.3390/ijms23031119 PMC8835029

[5] Tao S , Liang S , Zeng T , Yin D . Epigenetic modification‐related mechanisms of hepatocellular carcinoma resistance to immune checkpoint inhibition. Front Immunol. 2022;13:1043667. 36685594 10.3389/fimmu.2022.1043667 PMC9845774

[6] Alghazali MW , Al‐Hetty H , Ali ZMM , Saleh MM , Suleiman AA , Jalil AT . Non‐coding RNAs, another side of immune regulation during triple‐negative breast cancer. Pathol Res Pract. 2022;239:154132. 36183439 10.1016/j.prp.2022.154132

[7] Chen L , Deng J . Role of non‐coding RNA in immune microenvironment and anticancer therapy of gastric cancer. J Mol Med (Berl). 2022;100(12):1703‐1719. 36329206 10.1007/s00109-022-02264-6

[8] Yang F , Li J , Ge Q , et al. Non‐coding RNAs: emerging roles in the characterization of immune microenvironment and immunotherapy of prostate cancer. Biochem Pharmacol. 2023;214:115669. 37364622 10.1016/j.bcp.2023.115669

[9] Xia L , Oyang L , Lin J , et al. The cancer metabolic reprogramming and immune response. Mol Cancer. 2021;20(1):28. 33546704 10.1186/s12943-021-01316-8 PMC7863491

[10] Yan Y , Chang L , Tian H , et al. 1‐Pyrroline‐5‐carboxylate released by prostate Cancer cell inhibit T cell proliferation and function by targeting SHP1/cytochrome c oxidoreductase/ROS axis. J Immunother Cancer. 2018;6(1):148. 30545412 10.1186/s40425-018-0466-z PMC6291986

[11] Yang L , Chu Z , Liu M , et al. Amino acid metabolism in immune cells: essential regulators of the effector functions, and promising opportunities to enhance cancer immunotherapy. J Hematol Oncol. 2023;16(1):59. 37277776 10.1186/s13045-023-01453-1 PMC10240810

[12] Cui C , Wang J , Fagerberg E , et al. Neoantigen‐driven B cell and CD4 T follicular helper cell collaboration promotes anti‐tumor CD8 T cell responses. Cell. 2021;184(25):6101‐6118. e6113. 34852236 10.1016/j.cell.2021.11.007 PMC8671355

[13] Yu L , Feng R , Zhu L , et al. Promoting the activation of T cells with glycopolymer‐modified dendritic cells by enhancing cell interactions. Sci Adv. 2020;6(47):eabb6595. 33219021 10.1126/sciadv.abb6595 PMC7679162

[14] Balta E , Wabnitz GH , Samstag Y . Hijacked immune cells in the tumor microenvironment: molecular mechanisms of immunosuppression and cues to improve T cell‐based immunotherapy of solid tumors. Int J Mol Sci. 2021;22(11):5736. 34072260 10.3390/ijms22115736 PMC8199456

[15] Bai Z , Zhou Y , Ye Z , Xiong J , Lan H , Wang F . Tumor‐infiltrating lymphocytes in colorectal cancer: the fundamental indication and application on immunotherapy. Front Immunol. 2021;12:808964. 35095898 10.3389/fimmu.2021.808964 PMC8795622

[16] Paijens ST , Vledder A , de Bruyn M , Nijman HW . Tumor‐infiltrating lymphocytes in the immunotherapy era. Cell Mol Immunol. 2021;18(4):842‐859. 33139907 10.1038/s41423-020-00565-9 PMC8115290

[17] Jacquelot N , Tellier J , Nutt Sl , Belz Gt . Tertiary lymphoid structures and B lymphocytes in cancer prognosis and response to immunotherapies. Oncoimmunology. 2021;10(1):1900508. 33854820 10.1080/2162402X.2021.1900508 PMC8018489

[18] Dong Y , Wan Z , Gao X , Yang G , Liu L . Reprogramming immune cells for enhanced cancer immunotherapy: targets and strategies. Front Immunol. 2021;12:609762. 33968014 10.3389/fimmu.2021.609762 PMC8097044

[19] Gómez‐Aleza C , Nguyen B , Yoldi G , et al. Inhibition of RANK signaling in breast cancer induces an anti‐tumor immune response orchestrated by CD8+ T cells. Nat Commun. 2020;11(1):6335. 33303745 10.1038/s41467-020-20138-8 PMC7728758

[20] Finisguerra V , Dvorakova T , Formenti M , et al. Metformin improves cancer immunotherapy by directly rescuing tumor‐infiltrating CD8 T lymphocytes from hypoxia‐induced immunosuppression. J Immunother Cancer. 2023;11(5):e005719. 37147018 10.1136/jitc-2022-005719 PMC10163559

[21] Chu Y , Dai E , Li Y , et al. Pan‐cancer T cell atlas links a cellular stress response state to immunotherapy resistance. Nat Med. 2023;29(6):1550‐1562. 37248301 10.1038/s41591-023-02371-y PMC11421770

[22] Peng S , Hu P , Xiao YT , et al. Single‐cell analysis reveals EP4 as a target for restoring T‐cell infiltration and sensitizing prostate cancer to immunotherapy. Clin Cancer Res. 2022;28(3):552‐567. 34740924 10.1158/1078-0432.CCR-21-0299

[23] Luoma AM , Suo S , Wang Y , et al. Tissue‐resident memory and circulating T cells are early responders to pre‐surgical cancer immunotherapy. Cell. 2022;185(16):2918‐2935. e2929. 35803260 10.1016/j.cell.2022.06.018 PMC9508682

[24] Damei I , Trickovic T , Mami‐Chouaib F , Corgnac S . Tumor‐resident memory T cells as a biomarker of the response to cancer immunotherapy. Front Immunol. 2023;14:1205984. 37545498 10.3389/fimmu.2023.1205984 PMC10399960

[25] Sim MJW , Sun PD . T cell recognition of tumor neoantigens and insights into T cell immunotherapy. Front Immunol. 2022;13:833017. 35222422 10.3389/fimmu.2022.833017 PMC8867076

[26] Kristensen NP , Heeke C , Tvingsholm SA , et al. Neoantigen‐reactive CD8+ T cells affect clinical outcome of adoptive cell therapy with tumor‐infiltrating lymphocytes in melanoma. J Clin Invest. 2022;132(2):e150535. 34813506 10.1172/JCI150535 PMC8759789

[27] Meier SL , Satpathy AT , Wells DK . Bystander T cells in cancer immunology and therapy. Nat Cancer. 2022;3(2):143‐155. 35228747 10.1038/s43018-022-00335-8

[28] Liang Y , Tan Y , Guan B , et al. Single‐cell atlases link macrophages and CD8(+) T‐cell subpopulations to disease progression and immunotherapy response in urothelial carcinoma. Theranostics. 2022;12(18):7745‐7759. 36451860 10.7150/thno.77281 PMC9706581

[29] Trefny MP , Kirchhammer N , Auf der Maur P , et al. Deletion of SNX9 alleviates CD8 T cell exhaustion for effective cellular cancer immunotherapy. Nat Commun. 2023;14(1):86. 36732507 10.1038/s41467-022-35583-w PMC9895440

[30] Basu A , Ramamoorthi G , Albert G , et al. Differentiation and regulation of T(H) cells: a balancing act for cancer immunotherapy. Front Immunol. 2021;12:669474. 34012451 10.3389/fimmu.2021.669474 PMC8126720

[31] Barros MS , de Araújo ND , Magalhães‐Gama F , et al. γδ T cells for leukemia immunotherapy: new and expanding trends. Front Immunol. 2021;12:729085. 34630403 10.3389/fimmu.2021.729085 PMC8493128

[32] Liu X , Si F , Bagley D , et al. Blockades of effector T cell senescence and exhaustion synergistically enhance antitumor immunity and immunotherapy. J Immunother Cancer. 2022;10(10):e005020. 36192086 10.1136/jitc-2022-005020 PMC9535198

[33] Chen Y , Jia K , Sun Y , et al. Predicting response to immunotherapy in gastric cancer via multi‐dimensional analyses of the tumour immune microenvironment. Nat Commun. 2022;13(1):4851. 35982052 10.1038/s41467-022-32570-z PMC9388563

[34] Jiang S , Ding X , Wu Q , Cheng T , Xu M , Huang J . Identifying immune cells‐related phenotype to predict immunotherapy and clinical outcome in gastric cancer. Front Immunol. 2022;13:980986. 36032097 10.3389/fimmu.2022.980986 PMC9402937

[35] Qin Y , Lu F , Lyu K , Chang AE , Li Q . Emerging concepts regarding pro‐ and anti tumor properties of B cells in tumor immunity. Front Immunol. 2022;13:881427. 35967441 10.3389/fimmu.2022.881427 PMC9366002

[36] Guan L , Zhang Z , Gao T , et al. Depleting tumor infiltrating B cells to boost antitumor immunity with tumor immune‐microenvironment reshaped hybrid nanocage. ACS Nano. 2022;16(3):4263‐4277. 35179349 10.1021/acsnano.1c10283

[37] Yu Z , Li Y , Li Y , et al. Bufalin stimulates antitumor immune response by driving tumor‐infiltrating macrophage toward M1 phenotype in hepatocellular carcinoma. J Immunother Cancer. 2022;10(5):e004297. 35618286 10.1136/jitc-2021-004297 PMC9125767

[38] Anfray C , Mainini F , Digifico E , et al. Intratumoral combination therapy with poly(I:c) and resiquimod synergistically triggers tumor‐associated macrophages for effective systemic antitumoral immunity. J Immunother Cancer. 2021;9(9):e002408. 34531246 10.1136/jitc-2021-002408 PMC8449972

[39] Laureano RS , Sprooten J , Vanmeerbeerk I , et al. Trial watch: dendritic cell (DC)‐based immunotherapy for cancer. Oncoimmunology. 2022;11(1):2096363. 35800158 10.1080/2162402X.2022.2096363 PMC9255073

[40] Kießler M , Plesca I , Sommer U , et al. Tumor‐infiltrating plasmacytoid dendritic cells are associated with survival in human colon cancer. J Immunother Cancer. 2021;9(3):e001813. 33762320 10.1136/jitc-2020-001813 PMC7993360

[41] Creasy CA , Meng YJ , Forget MA , et al. Genomic correlates of outcome in tumor‐infiltrating lymphocyte therapy for metastatic melanoma. Clin Cancer Res. 2022;28(9):1911‐1924. 35190823 10.1158/1078-0432.CCR-21-1060 PMC9064946

[42] Huang L , Chen H , Xu Y , Chen J , Liu Z , Xu Q . Correlation of tumor‐infiltrating immune cells of melanoma with overall survival by immunogenomic analysis. Cancer Med. 2020;9(22):8444‐8456. 32931642 10.1002/cam4.3466 PMC7666744

[43] Dai Q , Wu W , Amei A , Yan X , Lu L , Wang Z . Regulation and characterization of tumor‐infiltrating immune cells in breast cancer. Int Immunopharmacol. 2021;90:107167. 33223469 10.1016/j.intimp.2020.107167 PMC7855363

[44] Nelson MA , Ngamcherdtrakul W , Luoh SW , Yantasee W . Prognostic and therapeutic role of tumor‐infiltrating lymphocyte subtypes in breast cancer. Cancer Metastasis Rev. 2021;40(2):519‐536. 33963482 10.1007/s10555-021-09968-0 PMC8424653

[45] Dieci MV , Miglietta F , Guarneri V . Immune infiltrates in breast cancer: recent updates and clinical implications. Cells. 2021;10(2):223. 33498711 10.3390/cells10020223 PMC7911608

[46] Chen Y , Zhao B , Wang X . Tumor infiltrating immune cells (TIICs) as a biomarker for prognosis benefits in patients with osteosarcoma. BMC Cancer. 2020;20(1):1022. 33087099 10.1186/s12885-020-07536-3 PMC7579940

[47] López‐Janeiro Á , Villalba‐Esparza M , Brizzi ME , et al. The association between the tumor immune microenvironments and clinical outcome in low‐grade, early‐stage endometrial cancer patients. J Pathol. 2022;258(4):426‐436. 36169332 10.1002/path.6012 PMC9828119

[48] Che Y , Luo Z , Zhang C , Sun N , Gao S , He J . Immune signature of tumor‐infiltrating immune cells predicts the prognosis and therapeutic effects in squamous cell carcinoma. Int Immunopharmacol. 2020;87:106802. 32745903 10.1016/j.intimp.2020.106802

[49] Dawson MA , Kouzarides T . Cancer epigenetics: from mechanism to therapy. Cell. 2012;150(1):12‐27. 22770212 10.1016/j.cell.2012.06.013

[50] Dziaman T , Gackowski D , Guz J , et al. Characteristic profiles of DNA epigenetic modifications in colon cancer and its predisposing conditions‐benign adenomas and inflammatory bowel disease. Clin Epigenetics. 2018;10:72. 29875879 10.1186/s13148-018-0505-0 PMC5977551

[51] Liang W , Zhao Y , Huang W , et al. Non‐invasive diagnosis of early‐stage lung cancer using high‐throughput targeted DNA methylation sequencing of circulating tumor DNA (ctDNA). Theranostics. 2019;9(7):2056‐2070. 31037156 10.7150/thno.28119 PMC6485294

[52] Yadav P , Subbarayalu P , Medina D , et al. M6A RNA methylation regulates histone ubiquitination to support cancer growth and progression. Cancer Res. 2022;82(10):1872‐1889. 35303054 10.1158/0008-5472.CAN-21-2106 PMC9336196

[53] Shi K , Yang S , Chen C , et al. RNA methylation‐mediated LINC01559 suppresses colorectal cancer progression by regulating the miR‐106b‐5p/PTEN axis. Int J Biol Sci. 2022;18(7):3048‐3065. 35541914 10.7150/ijbs.70630 PMC9066122

[54] Wang Y , Mao Y , Wang C , et al. RNA methylation‐related genes of m6A, m5C, and m1A predict prognosis and immunotherapy response in cervical cancer. Ann Med. 2023;55(1):2190618. 37042849 10.1080/07853890.2023.2190618 PMC10101678

[55] Li D , Li K , Zhang W , et al. The m6A/m5C/m1A regulated gene signature predicts the prognosis and correlates with the immune status of hepatocellular carcinoma. Front Immunol. 2022;13:918140. 35833147 10.3389/fimmu.2022.918140 PMC9272990

[56] Shao D , Li Y , Wu J , et al. An m6A/m5C/m1A/m7G‐related long non‐coding RNA signature to predict prognosis and immune features of glioma. Front Genet. 2022;13:903117. 35692827 10.3389/fgene.2022.903117 PMC9178125

[57] Audia JE , Campbell RM . Histone modifications and cancer. Cold Spring Harb Perspect Biol. 2016;8(4):a019521. 27037415 10.1101/cshperspect.a019521 PMC4817802

[58] Pan L , Feng F , Wu J , et al. Demethylzeylasteral targets lactate by inhibiting histone lactylation to suppress the tumorigenicity of liver cancer stem cells. Pharmacol Res. 2022;181:106270. 35605812 10.1016/j.phrs.2022.106270

[59] Chen TF , Hao HF , Zhang Y , et al. HBO1 induces histone acetylation and is important for non‐small cell lung cancer cell growth. Int J Biol Sci. 2022;18(8):3313‐3323. 35637972 10.7150/ijbs.72526 PMC9134900

[60] Deng Y , Gao J , Xu G , et al. HDAC6‐dependent deacetylation of AKAP12 dictates its ubiquitination and promotes colon cancer metastasis. Cancer Lett. 2022;549:215911. 36122629 10.1016/j.canlet.2022.215911

[61] Zhang Y , Liu Z , Yang X , et al. H3K27 acetylation activated‐COL6A1 promotes osteosarcoma lung metastasis by repressing STAT1 and activating pulmonary cancer‐associated fibroblasts. Theranostics. 2021;11(3):1473‐1492. 33391546 10.7150/thno.51245 PMC7738898

[62] Wattanathamsan O , Chantaravisoot N , Wongkongkathep P , et al. Inhibition of histone deacetylase 6 destabilizes ERK phosphorylation and suppresses cancer proliferation via modulation of the tubulin acetylation‐GRP78 interaction. J Biomed Sci. 2023;30(1):4. 36639650 10.1186/s12929-023-00898-3 PMC9838051

[63] Vatapalli R , Sagar V , Rodriguez Y , et al. Histone methyltransferase DOT1L coordinates AR and MYC stability in prostate cancer. Nat Commun. 2020;11(1):4153. 32814769 10.1038/s41467-020-18013-7 PMC7438336

[64] Lv J , Zhou Y , Zhou N , et al. Epigenetic modification of CSDE1 locus dictates immune recognition of nascent tumorigenic cells. Sci Transl Med. 2023;15(681):eabq6024. 36724242 10.1126/scitranslmed.abq6024

[65] Lukinović V , Hausmann S , Roth GS , et al. SMYD3 impedes small cell lung cancer sensitivity to alkylation damage through RNF113A methylation‐phosphorylation cross‐talk. Cancer Discov. 2022;12(9):2158‐2179. 35819319 10.1158/2159-8290.CD-21-0205 PMC9437563

[66] Hogg SJ , Beavis PA , Dawson MA , Johnstone RW . Targeting the epigenetic regulation of antitumour immunity. Nat Rev Drug Discov. 2020;19(11):776‐800. 32929243 10.1038/s41573-020-0077-5

[67] Villanueva L , Alvarez‐Errico D , Esteller M . The contribution of epigenetics to cancer immunotherapy. Trends Immunol. 2020;41(8):676‐691. 32622854 10.1016/j.it.2020.06.002

[68] Tough DF , Rioja I , Modis LK , Prinjha RK . Epigenetic regulation of T cell memory: recalling therapeutic implications. Trends Immunol. 2020;41(1):29‐45. 31813765 10.1016/j.it.2019.11.008

[69] Chiappinelli KB , Strissel PL , Desrichard A , et al. Inhibiting DNA methylation causes an interferon response in cancer via dsRNA including endogenous retroviruses. Cell. 2017;169(2):361. 10.1016/j.cell.2017.03.036 28388418

[70] Zebley CC , Abdelsamed HA , Ghoneim HE , et al. Proinflammatory cytokines promote TET2‐mediated DNA demethylation during CD8 T cell effector differentiation. Cell Rep. 2021;37(2):109796. 34644568 10.1016/j.celrep.2021.109796 PMC8593824

[71] Li HB , Tong J , Zhu S , et al. m(6)A mRNA methylation controls T cell homeostasis by targeting the IL‐7/STAT5/SOCS pathways. Nature. 2017;548(7667):338‐342. 28792938 10.1038/nature23450 PMC5729908

[72] Dong L , Chen C , Zhang Y , et al. The loss of RNA N(6)‐adenosine methyltransferase Mettl14 in tumor‐associated macrophages promotes CD8(+) T cell dysfunction and tumor growth. Cancer Cell. 2021;39(7):945‐957. e910. 34019807 10.1016/j.ccell.2021.04.016

[73] Zheng X , Sarode P , Weigert A , et al. The HDAC2‐SP1 axis orchestrates protumor macrophage polarization. Cancer Res. 2023;83(14):2345‐2357. 37205635 10.1158/0008-5472.CAN-22-1270

[74] Yang W , Feng Y , Zhou J , et al. A selective HDAC8 inhibitor potentiates antitumor immunity and efficacy of immune checkpoint blockade in hepatocellular carcinoma. Sci Transl Med. 2021;13(588):eaaz6804. 33827976 10.1126/scitranslmed.aaz6804

[75] Luda KM , Longo J , Kitchen‐Goosen SM , et al. Ketolysis drives CD8(+) T cell effector function through effects on histone acetylation. Immunity. 2023;56(9):2021‐2035. 37516105 10.1016/j.immuni.2023.07.002 PMC10528215

[76] Cho H , Son WC , Lee YS , et al. Differential effects of histone deacetylases on the expression of NKG2D ligands and NK cell‐mediated anticancer immunity in lung cancer cells. Molecules. 2021;26(13):3952. 34203519 10.3390/molecules26133952 PMC8271929

[77] Li L , Hao S , Gao M , et al. HDAC3 inhibition promotes antitumor immunity by enhancing CXCL10‐mediated chemotaxis and recruiting of immune cells. Cancer Immunol Res. 2023;11(5):657‐673. 36898011 10.1158/2326-6066.CIR-22-0317 PMC10155037

[78] Alanazi S , Rabelo Melo F , Pejler G . Tryptase regulates the epigenetic modification of core histones in mast cell leukemia cells. Front Immunol. 2021;12:804408. 34925389 10.3389/fimmu.2021.804408 PMC8674432

[79] Yu B , Luo F , Sun B , et al. KAT6A acetylation of SMAD3 regulates myeloid‐derived suppressor cell recruitment, metastasis, and immunotherapy in triple‐negative breast cancer. Adv Sci (Weinh). 2021;8(20):e2100014. 34392614 10.1002/advs.202100014 PMC8529494

[80] Piunti A , Shilatifard A . The roles of Polycomb repressive complexes in mammalian development and cancer. Nat Rev Mol Cell Biol. 2021;22(5):326‐345. 33723438 10.1038/s41580-021-00341-1

[81] Li J , Li Y , Cao Y , et al. Polycomb chromobox (Cbx) 7 modulates activation‐induced CD4+ T cell apoptosis. Arch Biochem Biophys. 2014;564:184‐188. 25449062 10.1016/j.abb.2014.10.004

[82] Ren L , Li Z , Zhou Y , et al. CBX4 promotes antitumor immunity by suppressing Pdcd1 expression in T cells. Mol Oncol. 2023;17(12):2694‐2708. 37691307 10.1002/1878-0261.13516 PMC10701776

[83] Jing R , Scarfo I , Najia MA , et al. EZH1 repression generates mature iPSC‐derived CAR T cells with enhanced antitumor activity. Cell Stem Cell. 2022;29(8):1181‐1196. e1186. 35931029 10.1016/j.stem.2022.06.014 PMC9386785

[84] Stairiker CJ , Pfister SX , Hendrickson E , et al. EZH2 inhibition compromises α4‐1BB‐mediated antitumor efficacy by reducing the survival and effector programming of CD8(+) T cells. Front Immunol. 2021;12:770080. 34925340 10.3389/fimmu.2021.770080 PMC8683156

[85] Ramakrishnan S , Granger V , Rak M , et al. Inhibition of EZH2 induces NK cell‐mediated differentiation and death in muscle‐invasive bladder cancer. Cell Death Differ. 2019;26(10):2100‐2114. 30692641 10.1038/s41418-019-0278-9 PMC6748105

[86] Chibaya L , Murphy KC , DeMarco KD , et al. EZH2 inhibition remodels the inflammatory senescence‐associated secretory phenotype to potentiate pancreatic cancer immune surveillance. Nat Cancer. 2023;4(6):872‐892. 37142692 10.1038/s43018-023-00553-8 PMC10516132

[87] DuPage M , Chopra G , Quiros J , et al. The chromatin‐modifying enzyme Ezh2 is critical for the maintenance of regulatory T cell identity after activation. Immunity. 2015;42(2):227‐238. 25680271 10.1016/j.immuni.2015.01.007 PMC4347854

[88] Yin J , Leavenworth JW , Li Y , et al. Ezh2 regulates differentiation and function of natural killer cells through histone methyltransferase activity. Proc Natl Acad Sci U S A. 2015;112(52):15988‐15993. 26668377 10.1073/pnas.1521740112 PMC4702963

[89] Wang D , Quiros J , Mahuron K , et al. Targeting EZH2 reprograms intratumoral regulatory T cells to enhance cancer immunity. Cell Rep. 2018;23(11):3262‐3274. 29898397 10.1016/j.celrep.2018.05.050 PMC6094952

[90] Liu F , Wu D , Wang X . Roles of CTCF in conformation and functions of chromosome. Semin Cell Dev Biol. 2019;90:168‐173. 30031212 10.1016/j.semcdb.2018.07.021

[91] Holwerda SJ , de Laat W . CTCF: the protein, the binding partners, the binding sites and their chromatin loops. Philos Trans R Soc Lond B Biol Sci. 2013;368(1620):20120369. 23650640 10.1098/rstb.2012.0369 PMC3682731

[92] Dehingia B , Milewska M , Janowski M , Pękowska A . CTCF shapes chromatin structure and gene expression in health and disease. EMBO Rep. 2022;23(9):e55146. 35993175 10.15252/embr.202255146 PMC9442299

[93] Shan Q , Zhu S , Chen X , et al. Tcf1‐CTCF cooperativity shapes genomic architecture to promote CD8(+) T cell homeostasis. Nat Immunol. 2022;23(8):1222‐1235. 35882936 10.1038/s41590-022-01263-6 PMC9579964

[94] Quon S , Yu B , Russ BE , et al. DNA architectural protein CTCF facilitates subset‐specific chromatin interactions to limit the formation of memory CD8(+) T cells. Immunity. 2023;56(5):959‐978. e910. 37040762 10.1016/j.immuni.2023.03.017 PMC10265493

[95] Yang B , Kim S , Jung WJ , et al. CTCF controls three‐dimensional enhancer network underlying the inflammatory response of bone marrow‐derived dendritic cells. Nat Commun. 2023;14(1):1277. 36882470 10.1038/s41467-023-36948-5 PMC9992691

[96] Stik G , Vidal E , Barrero M , et al. CTCF is dispensable for immune cell transdifferentiation but facilitates an acute inflammatory response. Nat Genet. 2020;52(7):655‐661. 32514124 10.1038/s41588-020-0643-0

[97] Hanahan D , Weinberg RA . Hallmarks of cancer: the next generation. Cell. 2011;144(5):646‐674. 21376230 10.1016/j.cell.2011.02.013

[98] Finley LWS . What is cancer metabolism? Cell. 2023;186(8):1670‐1688. 36858045 10.1016/j.cell.2023.01.038 PMC10106389

[99] Faubert B , Solmonson A , DeBerardinis RJ . Metabolic reprogramming and cancer progression. Science. 2020;368(6487):eaaw5473. 32273439 10.1126/science.aaw5473 PMC7227780

[100] Pavlova NN , Zhu J , Thompson CB . The hallmarks of cancer metabolism: still emerging. Cell Metab. 2022;34(3):355‐377. 35123658 10.1016/j.cmet.2022.01.007 PMC8891094

[101] Luo X , Zheng E , Wei L , et al. The fatty acid receptor CD36 promotes HCC progression through activating Src/PI3K/AKT axis‐dependent aerobic glycolysis. Cell Death Dis. 2021;12(4):328. 33771982 10.1038/s41419-021-03596-w PMC7997878

[102] Sun Z , Zhang R , Zhang X , et al. LINE‐1 promotes tumorigenicity and exacerbates tumor progression via stimulating metabolism reprogramming in non‐small cell lung cancer. Mol Cancer. 2022;21(1):147. 35842613 10.1186/s12943-022-01618-5 PMC9288060

[103] Jung J , Zeng H , Horng T . Metabolism as a guiding force for immunity. Nat Cell Biol. 2019;21(1):85‐93. 30602764 10.1038/s41556-018-0217-x

[104] Wu F , Fan J , He Y , et al. Single‐cell profiling of tumor heterogeneity and the microenvironment in advanced non‐small cell lung cancer. Nat Commun. 2021;12(1):2540. 33953163 10.1038/s41467-021-22801-0 PMC8100173

[105] Xiao Z , Dai Z , Locasale JW . Metabolic landscape of the tumor microenvironment at single cell resolution. Nat Commun. 2019;10(1):3763. 31434891 10.1038/s41467-019-11738-0 PMC6704063

[106] Leone RD , Powell JD . Metabolism of immune cells in cancer. Nat Rev Cancer. 2020;20(9):516‐531. 32632251 10.1038/s41568-020-0273-y PMC8041116

[107] Huang B , Song BL , Xu C . Cholesterol metabolism in cancer: mechanisms and therapeutic opportunities. Nat Metab. 2020;2(2):132‐141. 32694690 10.1038/s42255-020-0174-0

[108] Chen B , Gao A , Tu B , et al. Metabolic modulation via mTOR pathway and anti‐angiogenesis remodels tumor microenvironment using PD‐L1‐targeting codelivery. Biomaterials. 2020;255:120187. 32590192 10.1016/j.biomaterials.2020.120187

[109] Zhang B , Vogelzang A , Miyajima M , et al. B cell‐derived GABA elicits IL‐10(+) macrophages to limit anti‐tumour immunity. Nature. 2021;599(7885):471‐476. 34732892 10.1038/s41586-021-04082-1 PMC8599023

[110] Reinfeld BI , Madden MZ , Wolf MM , et al. Cell‐programmed nutrient partitioning in the tumour microenvironment. Nature. 2021;593(7858):282‐288. 33828302 10.1038/s41586-021-03442-1 PMC8122068

[111] Mu X , Xiang Z , Xu Y , et al. Glucose metabolism controls human γδ T‐cell‐mediated tumor immunosurveillance in diabetes. Cell Mol Immunol. 2022;19(8):944‐956. 35821253 10.1038/s41423-022-00894-x PMC9338301

[112] Watson MJ , Vignali PDA , Mullett SJ , et al. Metabolic support of tumour‐infiltrating regulatory T cells by lactic acid. Nature. 2021;591(7851):645‐651. 33589820 10.1038/s41586-020-03045-2 PMC7990682

[113] Kumagai S , Koyama S , Itahashi K , et al. Lactic acid promotes PD‐1 expression in regulatory T cells in highly glycolytic tumor microenvironments. Cancer Cell. 2022;40(2):201‐218. e209. 35090594 10.1016/j.ccell.2022.01.001

[114] Poznanski SM , Singh K , Ritchie TM , et al. Metabolic flexibility determines human NK cell functional fate in the tumor microenvironment. Cell Metab. 2021;33(6):1205‐1220. e1205. 33852875 10.1016/j.cmet.2021.03.023

[115] Feng Q , Liu Z , Yu X , et al. Lactate increases stemness of CD8 + T cells to augment anti‐tumor immunity. Nat Commun. 2022;13(1):4981. 36068198 10.1038/s41467-022-32521-8 PMC9448806

[116] Luu M , Riester Z , Baldrich A , et al. Microbial short‐chain fatty acids modulate CD8(+) T cell responses and improve adoptive immunotherapy for cancer. Nat Commun. 2021;12(1):4077. 34210970 10.1038/s41467-021-24331-1 PMC8249424

[117] Israr M , Lam F , DeVoti J , et al. PGE(2) expression by HPV6/11‐induced respiratory papillomas blocks NK cell activation in patients with recurrent respiratory papillomatosis. Eur J Immunol. 2023;53(4):e2250036. 36608264 10.1002/eji.202250036

[118] Pellegrini JM , Martin C , Morelli MP , et al. PGE2 displays immunosuppressive effects during human active tuberculosis. Sci Rep. 2021;11(1):13559. 34193890 10.1038/s41598-021-92667-1 PMC8245456

[119] Pi C , Jing P , Li B , et al. Reversing PD‐1 resistance in B16F10 cells and recovering tumour immunity using a COX2 inhibitor. Cancers (Basel). 2022;14(17):4134. 36077671 10.3390/cancers14174134 PMC9455073

[120] Thumkeo D , Punyawatthananukool S , Prasongtanakij S , et al. PGE(2)‐EP2/EP4 signaling elicits immunosuppression by driving the mregDC‐Treg axis in inflammatory tumor microenvironment. Cell Rep. 2022;39(10):110914. 35675777 10.1016/j.celrep.2022.110914

[121] Yamamichi K , Fukuda T , Sanui T , et al. Amelogenin induces M2 macrophage polarisation via PGE2/cAMP signalling pathway. Arch Oral Biol. 2017;83:241‐251. 28822800 10.1016/j.archoralbio.2017.08.005

[122] Cheng X , Tan X , Wang W , et al. Long‐chain acylcarnitines induce senescence of invariant natural killer T cells in hepatocellular carcinoma. Cancer Res. 2023;83(4):582‐594. 36512635 10.1158/0008-5472.CAN-22-2273

[123] Liu X , Hartman CL , Li L , et al. Reprogramming lipid metabolism prevents effector T cell senescence and enhances tumor immunotherapy. Sci Transl Med. 2021;13(587):eaaz6314. 33790024 10.1126/scitranslmed.aaz6314 PMC12040281

[124] Edwards DN , Ngwa VM , Raybuck AL , et al. Selective glutamine metabolism inhibition in tumor cells improves antitumor T lymphocyte activity in triple‐negative breast cancer. J Clin Invest. 2021;131(4):e140100. 33320840 10.1172/JCI140100 PMC7880417

[125] Huang M , Xiong D , Pan J , et al. Targeting glutamine metabolism to enhance immunoprevention of EGFR‐driven lung cancer. Adv Sci (Weinh). 2022;9(26):e2105885. 35861366 10.1002/advs.202105885 PMC9475521

[126] Leone RD , Zhao L , Englert JM , et al. Glutamine blockade induces divergent metabolic programs to overcome tumor immune evasion. Science. 2019;366(6468):1013‐1021. 31699883 10.1126/science.aav2588 PMC7023461

[127] Geiger R , Rieckmann JC , Wolf T , et al. l‐Arginine modulates T cell metabolism and enhances survival and anti‐tumor activity. Cell. 2016;167(3):829‐842. e813. 27745970 10.1016/j.cell.2016.09.031 PMC5075284

[128] Huang X , Sun T , Wang J , et al. Metformin reprograms tryptophan metabolism to stimulate CD8+ T‐cell function in colorectal cancer. Cancer Res. 2023;83(14):2358‐2371. 37195082 10.1158/0008-5472.CAN-22-3042

[129] Hung MH , Lee JS , Ma C , et al. Tumor methionine metabolism drives T‐cell exhaustion in hepatocellular carcinoma. Nat Commun. 2021;12(1):1455. 33674593 10.1038/s41467-021-21804-1 PMC7935900

[130] Sepich‐Poore GD , Zitvogel L , Straussman R , Hasty J , Wargo JA , Knight R . The microbiome and human cancer. Science. 2021;371(6536):eabc4552. 33766858 10.1126/science.abc4552 PMC8767999

[131] Schaupp L , Muth S , Rogell L , et al. Microbiota‐induced type I interferons instruct a poised basal state of dendritic cells. Cell. 2020;181(5):1080‐1096. e1019. 32380006 10.1016/j.cell.2020.04.022

[132] Winkler ES , Shrihari S . The intestinal microbiome restricts alphavirus infection and dissemination through a bile acid‐type I IFN signaling axis. Cell. 2020;182(4):901‐918. e918. 32668198 10.1016/j.cell.2020.06.029 PMC7483520

[133] Ma C , Han M , Heinrich B , et al. Gut microbiome‐mediated bile acid metabolism regulates liver cancer via NKT cells. Science. 2018;360(6391):eaan5931. 29798856 10.1126/science.aan5931 PMC6407885

[134] Overacre‐Delgoffe AE , Bumgarner HJ , Cillo AR , et al. Microbiota‐specific T follicular helper cells drive tertiary lymphoid structures and anti‐tumor immunity against colorectal cancer. Immunity. 2021;54(12):2812‐2824. e2814. 34861182 10.1016/j.immuni.2021.11.003 PMC8865366

[135] Pal S , Perrien DS , Yumoto T , et al. The microbiome restrains melanoma bone growth by promoting intestinal NK and Th1 cell homing to bone. J Clin Invest. 2022;132(12):e157340. 35503658 10.1172/JCI157340 PMC9197523

[136] He Y , Fu L , Li Y , et al. Gut microbial metabolites facilitate anticancer therapy efficacy by modulating cytotoxic CD8(+) T cell immunity. Cell Metab. 2021;33(5):988‐1000. e1007. 33761313 10.1016/j.cmet.2021.03.002

[137] Peng R , Liu S , You W , et al. Gastric microbiome alterations are associated with decreased CD8+ tissue‐resident memory T cells in the tumor microenvironment of gastric cancer. Cancer Immunol Res. 2022;10(10):1224‐1240. 35881964 10.1158/2326-6066.CIR-22-0107

[138] Xing C , Wang M , Ajibade AA , et al. Microbiota regulate innate immune signaling and protective immunity against cancer. Cell Host Microbe. 2021;29(6):959‐974. e957. 33894128 10.1016/j.chom.2021.03.016 PMC8192480

[139] Steinert EM , Vasan K , Chandel NS . Mitochondrial metabolism regulation of T cell‐mediated immunity. Annu Rev Immunol. 2021;39:395‐416. 33902315 10.1146/annurev-immunol-101819-082015 PMC10403253

[140] Wang Y , Li N , Zhang X , Horng T . Mitochondrial metabolism regulates macrophage biology. J Biol Chem. 2021;297(1):100904. 34157289 10.1016/j.jbc.2021.100904 PMC8294576

[141] Saha T , Dash C , Jayabalan R , et al. Intercellular nanotubes mediate mitochondrial trafficking between cancer and immune cells. Nat Nanotechnol. 2022;17(1):98‐106. 34795441 10.1038/s41565-021-01000-4 PMC10071558

[142] Gao Z , Li Y , Wang F , et al. Mitochondrial dynamics controls anti‐tumour innate immunity by regulating CHIP‐IRF1 axis stability. Nat Commun. 2017;8(1):1805. 29180626 10.1038/s41467-017-01919-0 PMC5703766

[143] Lötscher J , Martí ILAA , Kirchhammer N , et al. Magnesium sensing via LFA‐1 regulates CD8(+) T cell effector function. Cell. 2022;185(4):585‐602. e529. 35051368 10.1016/j.cell.2021.12.039

[144] Song J , Liu T , Yin Y , et al. The deubiquitinase OTUD1 enhances iron transport and potentiates host antitumor immunity. EMBO Rep. 2021;22(2):e51162. 33393230 10.15252/embr.202051162 PMC7857436

[145] Chen S , Cui W , Chi Z , et al. Tumor‐associated macrophages are shaped by intratumoral high potassium via Kir2.1. Cell Metab. 2022;34(11):1843‐1859. e1811. 36103895 10.1016/j.cmet.2022.08.016

[146] Lv M , Chen M , Zhang R , et al. Manganese is critical for antitumor immune responses via cGAS‐STING and improves the efficacy of clinical immunotherapy. Cell Res. 2020;30(11):966‐979. 32839553 10.1038/s41422-020-00395-4 PMC7785004

[147] Cen D , Ge Q , Xie C , et al. ZnS@BSA nanoclusters potentiate efficacy of cancer immunotherapy. Adv Mater. 2021;33(49):e2104037. 34622500 10.1002/adma.202104037

[148] Li Q , Chao Y , Liu B , et al. Disulfiram loaded calcium phosphate nanoparticles for enhanced cancer immunotherapy. Biomaterials. 2022;291:121880. 36334355 10.1016/j.biomaterials.2022.121880

[149] Zheng P , Ding B , Jiang Z , et al. Ultrasound‐augmented mitochondrial calcium ion overload by calcium nanomodulator to induce immunogenic cell death. Nano Lett. 2021;21(5):2088‐2093. 33596078 10.1021/acs.nanolett.0c04778

[150] Shyer JA , Flavell RA , Bailis W . Metabolic signaling in T cells. Cell Res. 2020;30(8):649‐659. 32709897 10.1038/s41422-020-0379-5 PMC7395146

[151] Yang F , Wang T , Du P , Fan H , Dong X , Guo H . M2 bone marrow‐derived macrophage‐derived exosomes shuffle microRNA‐21 to accelerate immune escape of glioma by modulating PEG3. Cancer Cell Int. 2020;20:93. 32231463 10.1186/s12935-020-1163-9 PMC7099792

[152] Du T , Yang CL , Ge MR , et al. M1 macrophage derived exosomes aggravate experimental autoimmune neuritis via modulating Th1 response. Front Immunol. 2020;11:1603. 32793234 10.3389/fimmu.2020.01603 PMC7390899

[153] Pitt JM , André F , Amigorena S , et al. Dendritic cell‐derived exosomes for cancer therapy. J Clin Invest. 2016;126(4):1224‐1232. 27035813 10.1172/JCI81137 PMC4811123

[154] Viaud S , Terme M , Flament C , et al. Dendritic cell‐derived exosomes promote natural killer cell activation and proliferation: a role for NKG2D ligands and IL‐15Ralpha. PLoS One. 2009;4(3):e4942. 19319200 10.1371/journal.pone.0004942 PMC2657211

[155] Xiong J , Chi H , Yang G , et al. Revolutionizing anti‐tumor therapy: unleashing the potential of B cell‐derived exosomes. Front Immunol. 2023;14:1188760. 37342327 10.3389/fimmu.2023.1188760 PMC10277631

[156] Klinker MW , Lizzio V , Reed TJ , Fox DA , Lundy SK . Human B cell‐derived lymphoblastoid cell lines constitutively produce fas ligand and secrete MHCII(+)FasL(+) killer exosomes. Front Immunol. 2014;5:144. 24765093 10.3389/fimmu.2014.00144 PMC3980107

[157] Tung SL , Boardman DA , Sen M , et al. Regulatory T cell‐derived extracellular vesicles modify dendritic cell function. Sci Rep. 2018;8(1):6065. 29666503 10.1038/s41598-018-24531-8 PMC5904112

[158] Zhou J , Li X , Wu X , et al. Exosomes released from tumor‐associated macrophages transfer miRNAs that induce a Treg/Th17 cell imbalance in epithelial ovarian cancer. Cancer Immunol Res. 2018;6(12):1578‐1592. 30396909 10.1158/2326-6066.CIR-17-0479

[159] Skokos D , Botros HG , Demeure C , et al. Mast cell‐derived exosomes induce phenotypic and functional maturation of dendritic cells and elicit specific immune responses in vivo. J Immunol. 2003;170(6):3037‐3045. 12626558 10.4049/jimmunol.170.6.3037

[160] Fabbri M . Natural killer cell‐derived vesicular miRNAs: a new anticancer approach? Cancer Res. 2020;80(1):17‐22. 31672842 10.1158/0008-5472.CAN-19-1450 PMC6942618

[161] Pramanik A , Bhattacharyya S . Myeloid derived suppressor cells and innate immune system interaction in tumor microenvironment. Life Sci. 2022;305:120755. 35780842 10.1016/j.lfs.2022.120755

[162] Franklin RA , Liao W , Sarkar A , et al. The cellular and molecular origin of tumor‐associated macrophages. Science. 2014;344(6186):921‐925. 24812208 10.1126/science.1252510 PMC4204732

[163] Sinha P , Clements VK , Bunt SK , Albelda SM , Ostrand‐Rosenberg S . Cross‐talk between myeloid‐derived suppressor cells and macrophages subverts tumor immunity toward a type 2 response. J Immunol. 2007;179(2):977‐983. 17617589 10.4049/jimmunol.179.2.977

[164] Thibodeau J , Bourgeois‐Daigneault MC , Huppé G , et al. Interleukin‐10‐induced MARCH1 mediates intracellular sequestration of MHC class II in monocytes. Eur J Immunol. 2008;38(5):1225‐1230. 18389477 10.1002/eji.200737902 PMC2759377

[165] Li H , Han Y , Guo Q , Zhang M , Cao X . Cancer‐expanded myeloid‐derived suppressor cells induce anergy of NK cells through membrane‐bound TGF‐beta 1. J Immunol. 2009;182(1):240‐249. 19109155 10.4049/jimmunol.182.1.240

[166] Joshi S , Sharabi A . Targeting myeloid‐derived suppressor cells to enhance natural killer cell‐based immunotherapy. Pharmacol Ther. 2022;235:108114. 35122833 10.1016/j.pharmthera.2022.108114 PMC9189042

[167] Gabrilovich DI . Myeloid‐Derived Suppressor Cells. Cancer Immunol Res. 2017;5(1):3‐8. 28052991 10.1158/2326-6066.CIR-16-0297 PMC5426480

[168] Zhang J , Han X , Hu X , et al. IDO1 impairs NK cell cytotoxicity by decreasing NKG2D/NKG2DLs via promoting miR‐18a. Mol Immunol. 2018;103:144‐155. 30268986 10.1016/j.molimm.2018.09.011

[169] Zhao H , Teng D , Yang L , et al. Myeloid‐derived itaconate suppresses cytotoxic CD8(+) T cells and promotes tumour growth. Nat Metab. 2022;4(12):1660‐1673. 36376563 10.1038/s42255-022-00676-9 PMC10593361

[170] Grazioli P , Orlando A , Giordano N , et al. Notch‐signaling deregulation induces myeloid‐derived suppressor cells in T‐Cell acute lymphoblastic leukemia. Front Immunol. 2022;13:809261. 35444651 10.3389/fimmu.2022.809261 PMC9013886

[171] Dar AA , Patil RS , Pradhan TN , Chaukar DA , D'Cruz AK , Chiplunkar SV . Myeloid‐derived suppressor cells impede T cell functionality and promote Th17 differentiation in oral squamous cell carcinoma. Cancer Immunol Immunother. 2020;69(6):1071‐1086. 32103293 10.1007/s00262-020-02523-w PMC11027600

[172] Haist M , Stege H , Grabbe S , Bros M . The functional crosstalk between myeloid‐derived suppressor cells and regulatory T cells within the immunosuppressive tumor microenvironment. Cancers (Basel). 2021;13(2):210. 33430105 10.3390/cancers13020210 PMC7827203

[173] Ostrand‐Rosenberg S , Sinha P , Beury DW , Clements VK . Cross‐talk between myeloid‐derived suppressor cells (MDSC), macrophages, and dendritic cells enhances tumor‐induced immune suppression. Semin Cancer Biol. 2012;22(4):275‐281. 22313874 10.1016/j.semcancer.2012.01.011 PMC3701942

[174] Garaud S , Dieu‐Nosjean MC , Willard‐Gallo K . T follicular helper and B cell crosstalk in tertiary lymphoid structures and cancer immunotherapy. Nat Commun. 2022;13(1):2259. 35473931 10.1038/s41467-022-29753-z PMC9043192

[175] Poschke I , Mao Y , Adamson L , Salazar‐Onfray F , Masucci G , Kiessling R . Myeloid‐derived suppressor cells impair the quality of dendritic cell vaccines. Cancer Immunol Immunother. 2012;61(6):827‐838. 22080405 10.1007/s00262-011-1143-y PMC11028420

[176] Ugolini A , Tyurin VA , Tyurina YY , et al. Polymorphonuclear myeloid‐derived suppressor cells limit antigen cross‐presentation by dendritic cells in cancer. JCI Insight. 2020;5(15):e138581. 32584791 10.1172/jci.insight.138581 PMC7455061

[177] Kouketsu A , Haruka S , Kuroda K , et al. Myeloid‐derived suppressor cells and plasmacytoid dendritic cells are associated with oncogenesis of oral squamous cell carcinoma. J Oral Pathol Med. 2023;52(1):9‐19. 36380437 10.1111/jop.13386 PMC10108148

[178] Mun JY , Leem SH , Lee JH , Kim HS . Dual relationship between stromal cells and immune cells in the tumor microenvironment. Front Immunol. 2022;13:864739. 35464435 10.3389/fimmu.2022.864739 PMC9019709

[179] Ksiazkiewicz M , Gottfried E , Kreutz M , Mack M , Hofstaedter F , Kunz‐Schughart LA . Importance of CCL2‐CCR2A/2B signaling for monocyte migration into spheroids of breast cancer‐derived fibroblasts. Immunobiology. 2010;215(9‐10):737‐747. 20605053 10.1016/j.imbio.2010.05.019

[180] Gok Yavuz B , Gunaydin G , Gedik ME , et al. Cancer associated fibroblasts sculpt tumour microenvironment by recruiting monocytes and inducing immunosuppressive PD‐1(+) TAMs. Sci Rep. 2019;9(1):3172. 30816272 10.1038/s41598-019-39553-z PMC6395633

[181] Cheng Y , Li H , Deng Y , et al. Cancer‐associated fibroblasts induce PDL1+ neutrophils through the IL6‐STAT3 pathway that foster immune suppression in hepatocellular carcinoma. Cell Death Dis. 2018;9(4):422. 29556041 10.1038/s41419-018-0458-4 PMC5859264

[182] Qian L , Tang Z , Yin S , et al. Fusion of dendritic cells and cancer‐associated fibroblasts for activation of anti‐tumor cytotoxic T lymphocytes. J Biomed Nanotechnol. 2018;14(10):1826‐1835. 30041728 10.1166/jbn.2018.2616

[183] Sung E , Ko M , Won JY , et al. LAG‐3xPD‐L1 bispecific antibody potentiates antitumor responses of T cells through dendritic cell activation. Mol Ther. 2022;30(8):2800‐2816. 35526096 10.1016/j.ymthe.2022.05.003 PMC9372323

[184] Liu L , Chen J , Bae J , et al. Rejuvenation of tumour‐specific T cells through bispecific antibodies targeting PD‐L1 on dendritic cells. Nat Biomed Eng. 2021;5(11):1261‐1273. 34725504 10.1038/s41551-021-00800-2 PMC9499378

[185] Shapir Itai Y , Barboy O , Salomon R , et al. Bispecific dendritic‐T cell engager potentiates anti‐tumor immunity. Cell. 2024;187(2):375‐389. e318. 38242085 10.1016/j.cell.2023.12.011

[186] Hossain MA , Liu G , Dai B , et al. Reinvigorating exhausted CD8(+) cytotoxic T lymphocytes in the tumor microenvironment and current strategies in cancer immunotherapy. Med Res Rev. 2021;41(1):156‐201. 32844499 10.1002/med.21727

[187] Leone RD , Powell JD . Fueling the revolution: targeting metabolism to enhance immunotherapy. Cancer Immunol Res. 2021;9(3):255‐260. 33648947 10.1158/2326-6066.CIR-20-0791 PMC8240594

[188] Li X , Wenes M , Romero P , Huang SC , Fendt SM , Ho PC . Navigating metabolic pathways to enhance antitumour immunity and immunotherapy. Nat Rev Clin Oncol. 2019;16(7):425‐441. 30914826 10.1038/s41571-019-0203-7

[189] Gu M , Zhou X , Sohn JH , et al. NF‐κB‐inducing kinase maintains T cell metabolic fitness in antitumor immunity. Nat Immunol. 2021;22(2):193‐204. 33398181 10.1038/s41590-020-00829-6 PMC7855506

[190] Sievers C , Craveiro M , Friedman J , et al. Phenotypic plasticity and reduced tissue retention of exhausted tumor‐infiltrating T cells following neoadjuvant immunotherapy in head and neck cancer. Cancer Cell. 2023;41(5):887‐902. e885. 37059104 10.1016/j.ccell.2023.03.014 PMC10175181

[191] Wang Y , Wang F , Wang L , et al. NAD(+) supplement potentiates tumor‐killing function by rescuing defective TUB‐mediated NAMPT transcription in tumor‐infiltrated T cells. Cell Rep. 2021;36(6):109516. 34380043 10.1016/j.celrep.2021.109516

[192] Wang X , Li B , Kim YJ , et al. Targeting monoamine oxidase A for T cell‐based cancer immunotherapy. Sci Immunol. 2021;6(59):eabh2383. 33990379 10.1126/sciimmunol.abh2383

[193] Li Q , Xiang M . Metabolic reprograming of MDSCs within tumor microenvironment and targeting for cancer immunotherapy. Acta Pharmacol Sin. 2022;43(6):1337‐1348. 34561553 10.1038/s41401-021-00776-4 PMC9160034

[194] Gao A , Liu X , Lin W , et al. Tumor‐derived ILT4 induces T cell senescence and suppresses tumor immunity. J Immunother Cancer. 2021;9(3):e001536. 33653799 10.1136/jitc-2020-001536 PMC7929805

[195] Liu J , Shen H , Gu W , et al. Prediction of prognosis, immunogenicity and efficacy of immunotherapy based on glutamine metabolism in lung adenocarcinoma. Front Immunol. 2022;13:960738. 36032135 10.3389/fimmu.2022.960738 PMC9403193

[196] Yoshikawa T , Wu Z , Inoue S , et al. Genetic ablation of PRDM1 in antitumor T cells enhances therapeutic efficacy of adoptive immunotherapy. Blood. 2022;139(14):2156‐2172. 34861037 10.1182/blood.2021012714

[197] Zhou J , Kryczek I , Li S , et al. The ubiquitin ligase MDM2 sustains STAT5 stability to control T cell‐mediated antitumor immunity. Nat Immunol. 2021;22(4):460‐470. 33767425 10.1038/s41590-021-00888-3 PMC8026726

[198] Zou Q , Wang X , Ren D , et al. DNA methylation‐based signature of CD8+ tumor‐infiltrating lymphocytes enables evaluation of immune response and prognosis in colorectal cancer. J Immunother Cancer. 2021;9(9):e002671. 34548385 10.1136/jitc-2021-002671 PMC8458312

[199] Yu M , Liu X , Xu H , et al. Comprehensive evaluation of the m(6)A regulator prognostic risk score in the prediction of immunotherapy response in clear cell renal cell carcinoma. Front Immunol. 2022;13:818120. 35784363 10.3389/fimmu.2022.818120 PMC9248360

[200] Dong H , Xie C , Yao Z , et al. PTPRO‐related CD8(+) T‐cell signatures predict prognosis and immunotherapy response in patients with breast cancer. Front Immunol. 2022;13:947841. 36003382 10.3389/fimmu.2022.947841 PMC9393709

[201] Tanagala KKK , Morin‐Baxter J , Carvajal R , et al. SP140 inhibits STAT1 signaling, induces IFN‐γ in tumor‐associated macrophages, and is a predictive biomarker of immunotherapy response. J Immunother Cancer. 2022;10(12):e005088. 36600652 10.1136/jitc-2022-005088 PMC9748993

[202] Petitprez F , Meylan M , de Reyniès A , Sautès‐Fridman C , Fridman WH . The tumor microenvironment in the response to immune checkpoint blockade therapies. Front Immunol. 2020;11:784. 32457745 10.3389/fimmu.2020.00784 PMC7221158

[203] Jaiswal A , Verma A , Dannenfelser R , et al. An activation to memory differentiation trajectory of tumor‐infiltrating lymphocytes informs metastatic melanoma outcomes. Cancer Cell. 2022;40(5):524‐544. e525. 35537413 10.1016/j.ccell.2022.04.005 PMC9122099

[204] Wei SC , Duffy CR , Allison JP . Fundamental mechanisms of immune checkpoint blockade therapy. Cancer Discov. 2018;8(9):1069‐1086. 30115704 10.1158/2159-8290.CD-18-0367

[205] Tian X , Ning Q , Yu J , Tang S . T‐cell immunoglobulin and ITIM domain in cancer immunotherapy: a focus on tumor‐infiltrating regulatory T cells. Mol Immunol. 2022;147:62‐70. 35504059 10.1016/j.molimm.2022.04.014

[206] Tay C , Tanaka A , Sakaguchi S . Tumor‐infiltrating regulatory T cells as targets of cancer immunotherapy. Cancer Cell. 2023;41(3):450‐465. 36917950 10.1016/j.ccell.2023.02.014

[207] Stanczak MA , Läubli H . Siglec receptors as new immune checkpoints in cancer. Mol Aspects Med. 2023;90:101112. 35948467 10.1016/j.mam.2022.101112

[208] Wang J , Sun J , Liu LN , et al. Siglec‐15 as an immune suppressor and potential target for normalization cancer immunotherapy. Nat Med. 2019;25(4):656‐666. 30833750 10.1038/s41591-019-0374-x PMC7175920

[209] Xu JX , Maher VE , Zhang L , et al. FDA approval summary: nivolumab in advanced renal cell carcinoma after anti‐angiogenic therapy and exploratory predictive biomarker analysis. Oncologist. 2017;22(3):311‐317. 28232599 10.1634/theoncologist.2016-0476 PMC5344649

[210] Kazandjian D , Suzman DL , Blumenthal G , et al. FDA approval summary: nivolumab for the treatment of metastatic non‐small cell lung cancer with progression on or after platinum‐based chemotherapy. Oncologist. 2016;21(5):634‐642. 26984449 10.1634/theoncologist.2015-0507 PMC4861371

[211] Kasamon YL , de Claro RA , Wang Y , Shen YL , Farrell AT , Pazdur R . FDA approval summary: nivolumab for the treatment of relapsed or progressive classical hodgkin lymphoma. Oncologist. 2017;22(5):585‐591. 28438889 10.1634/theoncologist.2017-0004 PMC5423515

[212] Horiba MN , Casak SJ , Mishra‐Kalyani PS , et al. FDA approval summary: nivolumab for the adjuvant treatment of adults with completely resected esophageal/gastroesophageal junction cancer and residual pathologic disease. Clin Cancer Res. 2022;28(24):5244‐5248. 35960160 10.1158/1078-0432.CCR-22-0617 PMC9771915

[213] Shah M , Osgood CL , Amatya AK , et al. FDA approval summary: pembrolizumab for neoadjuvant and adjuvant treatment of patients with high‐risk early‐stage triple‐negative breast cancer. Clin Cancer Res. 2022;28(24):5249‐5253. 35925043 10.1158/1078-0432.CCR-22-1110

[214] Pai‐Scherf L , Blumenthal GM , Li H , et al. FDA Approval summary: pembrolizumab for treatment of metastatic non‐small cell lung cancer: first‐line therapy and beyond. Oncologist. 2017;22(11):1392‐1399. 28835513 10.1634/theoncologist.2017-0078 PMC5679831

[215] Chuk MK , Chang JT , Theoret MR , et al. FDA approval summary: accelerated approval of pembrolizumab for second‐line treatment of metastatic melanoma. Clin Cancer Res. 2017;23(19):5666‐5670. 28235882 10.1158/1078-0432.CCR-16-0663

[216] Suzman DL , Agrawal S , Ning YM , et al. FDA approval summary: atezolizumab or pembrolizumab for the treatment of patients with advanced urothelial carcinoma ineligible for cisplatin‐containing chemotherapy. Oncologist. 2019;24(4):563‐569. 30541754 10.1634/theoncologist.2018-0084 PMC6459239

[217] Mathieu LN , Larkins E , Sinha AK , et al. FDA approval summary: atezolizumab as adjuvant treatment following surgical resection and platinum‐based chemotherapy for stage II to IIIA NSCLC. Clin Cancer Res. 2023;29(16):2973‐2978. 36951523 10.1158/1078-0432.CCR-22-3699 PMC10440223

[218] Casak SJ , Donoghue M , Fashoyin‐Aje L , et al. FDA approval summary: atezolizumab plus bevacizumab for the treatment of patients with advanced unresectable or metastatic hepatocellular carcinoma. Clin Cancer Res. 2021;27(7):1836‐1841. 33139264 10.1158/1078-0432.CCR-20-3407

[219] Powles T , Park SH , Voog E , et al. Avelumab maintenance therapy for advanced or metastatic urothelial carcinoma. N Engl J Med. 2020;383(13):1218‐1230. 32945632 10.1056/NEJMoa2002788

[220] D'Angelo SP , Lebbé C , Mortier L , et al. First‐line avelumab in a cohort of 116 patients with metastatic Merkel cell carcinoma (JAVELIN Merkel 200): primary and biomarker analyses of a phase II study. J Immunother Cancer. 2021;9(7):e002646. 34301810 10.1136/jitc-2021-002646 PMC8311489

[221] Keam SJ . Tremelimumab: first approval. Drugs. 2023;83(1):93‐102. 36571670 10.1007/s40265-022-01827-8

[222] Cameron F , Whiteside G , Perry C . Ipilimumab: first global approval. Drugs. 2011;71(8):1093‐1104. 21668044 10.2165/11594010-000000000-00000

[223] Blum SM , Rouhani SJ , Sullivan RJ . Effects of immune‐related adverse events (irAEs) and their treatment on antitumor immune responses. Immunol Rev. 2023;318(1):167‐178. 37578634 10.1111/imr.13262

[224] Tong J , Kartolo A , Yeung C , Hopman W , Baetz T . Long‐term toxicities of immune checkpoint inhibitor (ICI) in melanoma patients. Curr Oncol. 2022;29(10):7953‐7963. 36290906 10.3390/curroncol29100629 PMC9600354

[225] Kantoff PW , Higano CS , Shore ND , et al. Sipuleucel‐T immunotherapy for castration‐resistant prostate cancer. N Engl J Med. 2010;363(5):411‐422. 20818862 10.1056/NEJMoa1001294

[226] Brower V . Approval of provenge seen as first step for cancer treatment vaccines. J Natl Cancer Inst. 2010;102(15):1108‐1110. 20668267 10.1093/jnci/djq295

[227] Maude SL , Laetsch TW , Buechner J , et al. Tisagenlecleucel in children and young adults with B‐cell lymphoblastic leukemia. N Engl J Med. 2018;378(5):439‐448. 29385370 10.1056/NEJMoa1709866 PMC5996391

[228] Bach PB , Giralt SA , Saltz LB . FDA approval of tisagenlecleucel: promise and complexities of a $475 000 cancer drug. Jama. 2017;318(19):1861‐1862. 28975266 10.1001/jama.2017.15218

[229] Neelapu SS , Locke FL , Bartlett NL , et al. Axicabtagene ciloleucel CAR T‐cell therapy in refractory large B‐cell lymphoma. N Engl J Med. 2017;377(26):2531‐2544. 29226797 10.1056/NEJMoa1707447 PMC5882485

[230] FDA approves second CAR T‐cell therapy. Cancer Discov. 2018;8(1):5‐6. 29113977 10.1158/2159-8290.CD-NB2017-155

[231] Frey NV . Approval of brexucabtagene autoleucel for adults with relapsed and refractory acute lymphocytic leukemia. Blood. 2022;140(1):11‐15. 35507688 10.1182/blood.2021014892

[232] Abramson JS , Palomba ML , Gordon LI , et al. Lisocabtagene maraleucel for patients with relapsed or refractory large B‐cell lymphomas (TRANSCEND NHL 001): a multicentre seamless design study. Lancet. 2020;396(10254):839‐852. 32888407 10.1016/S0140-6736(20)31366-0

[233] Sun D , Liu J , Zhou H , et al. Classification of tumor immune microenvironment according to programmed death‐ligand 1 expression and immune infiltration predicts response to immunotherapy plus chemotherapy in advanced patients with NSCLC. J Thorac Oncol. 2023;18(7):869‐881. 36948245 10.1016/j.jtho.2023.03.012

[234] Rohaan MW , Borch TH , van den Berg JH , et al. Tumor‐infiltrating lymphocyte therapy or ipilimumab in advanced melanoma. N Engl J Med. 2022;387(23):2113‐2125. 36477031 10.1056/NEJMoa2210233

[235] Hall MS , Mullinax JE , Cox CA , et al. Combination nivolumab, CD137 agonism, and adoptive cell therapy with tumor‐infiltrating lymphocytes for patients with metastatic melanoma. Clin Cancer Res. 2022;28(24):5317‐5329. 36215121 10.1158/1078-0432.CCR-22-2103 PMC10324027

[236] Liang YJ , Chen QY , Xu JX , et al. A phase II randomised controlled trial of adjuvant tumour‐infiltrating lymphocytes for pretreatment Epstein‐Barr virus DNA‐selected high‐risk nasopharyngeal carcinoma patients. Eur J Cancer. 2023;191:112965. 37540921 10.1016/j.ejca.2023.112965

[237] Mailankody S , Devlin SM , Landa J , et al. GPRC5D‐targeted CAR T cells for myeloma. N Engl J Med. 2022;387(13):1196‐1206. 36170501 10.1056/NEJMoa2209900 PMC10309537

[238] Mailankody S , Matous JV , Chhabra S , et al. Allogeneic BCMA‐targeting CAR T cells in relapsed/refractory multiple myeloma: phase 1 UNIVERSAL trial interim results. Nat Med. 2023;29(2):422‐429. 36690811 10.1038/s41591-022-02182-7

[239] Gill S , Vides V , Frey NV , et al. Anti‐CD19 CAR T cells in combination with ibrutinib for the treatment of chronic lymphocytic leukemia. Blood Adv. 2022;6(21):5774‐5785. 35349631 10.1182/bloodadvances.2022007317 PMC9647791

[240] Park JH , Nath K , Devlin SM , et al. CD19 CAR T‐cell therapy and prophylactic anakinra in relapsed or refractory lymphoma: phase 2 trial interim results. Nat Med. 2023;29(7):1710‐1717. 37400640 10.1038/s41591-023-02404-6 PMC11462637

[241] Pan K , Farrukh H , Chittepu V , Xu H , Pan CX , Zhu Z . CAR race to cancer immunotherapy: from CAR T, CAR NK to CAR macrophage therapy. J Exp Clin Cancer Res. 2022;41(1):119. 35361234 10.1186/s13046-022-02327-z PMC8969382

[242] Chen HX , Song M , Maecker HT , et al. Network for biomarker immunoprofiling for cancer immunotherapy: cancer immune monitoring and analysis centers and cancer immunologic data commons (CIMAC‐CIDC). Clin Cancer Res. 2021;27(18):5038‐5048. 33419780 10.1158/1078-0432.CCR-20-3241 PMC8491462

[243] Bai Y , Xie T , Wang Z , et al. Efficacy and predictive biomarkers of immunotherapy in Epstein‐Barr virus‐associated gastric cancer. J Immunother Cancer. 2022;10(3):e004080. 35241494 10.1136/jitc-2021-004080 PMC8896035

[244] Sadeghirad H , Liu N , Monkman J , et al. Compartmentalized spatial profiling of the tumor microenvironment in head and neck squamous cell carcinoma identifies immune checkpoint molecules and tumor necrosis factor receptor superfamily members as biomarkers of response to immunotherapy. Front Immunol. 2023;14:1135489. 37153589 10.3389/fimmu.2023.1135489 PMC10154785

[245] Zhou X , Cao J , Topatana W , et al. Evaluation of PD‐L1 as a biomarker for immunotherapy for hepatocellular carcinoma: systematic review and meta‐analysis. Immunotherapy. 2023;15(5):353‐365. 36852452 10.2217/imt-2022-0168

[246] Marcos Rubio A , Everaert C , Van Damme E , De Preter K , Vermaelen K . Circulating immune cell dynamics as outcome predictors for immunotherapy in non‐small cell lung cancer. J Immunother Cancer. 2023;11(8):e007023. 37536935 10.1136/jitc-2023-007023 PMC10401220

[247] Sivapalan L , Murray JC , Canzoniero JV , et al. Liquid biopsy approaches to capture tumor evolution and clinical outcomes during cancer immunotherapy. J Immunother Cancer. 2023;11(1):e005924. 36657818 10.1136/jitc-2022-005924 PMC9853269

[248] Chalfin HJ , Pramparo T , Mortazavi A , et al. Circulating tumor cell subtypes and T‐cell populations as prognostic biomarkers to combination immunotherapy in patients with metastatic genitourinary cancer. Clin Cancer Res. 2021;27(5):1391‐1398. 33262136 10.1158/1078-0432.CCR-20-2891 PMC7925349

[249] Duan L , Liu X , Luo Z , et al. G‐protein subunit gamma 4 as a potential biomarker for predicting the response of chemotherapy and immunotherapy in bladder cancer. Genes (Basel). 2022;13(4):693. 35456499 10.3390/genes13040693 PMC9027884

[250] Kim ES , Velcheti V , Mekhail T , et al. Blood‐based tumor mutational burden as a biomarker for atezolizumab in non‐small cell lung cancer: the phase 2 B‐F1RST trial. Nat Med. 2022;28(5):939‐945. 35422531 10.1038/s41591-022-01754-x PMC9117143

[251] Shen R , Postow MA , Adamow M , et al. LAG‐3 expression on peripheral blood cells identifies patients with poorer outcomes after immune checkpoint blockade. Sci Transl Med. 2021;13(608):eabf5107. 34433638 10.1126/scitranslmed.abf5107 PMC9254663

[252] Signorelli D , Ghidotti P , Proto C , et al. Circulating CD81‐expressing extracellular vesicles as biomarkers of response for immune‐checkpoint inhibitors in advanced NSCLC. Front Immunol. 2022;13:987639. 36203609 10.3389/fimmu.2022.987639 PMC9530186

[253] Clevers MR , Kastelijn EA , Peters BJM , Kelder H , Schramel F . Evaluation of serum biomarker CEA and Ca‐125 as immunotherapy response predictors in metastatic non‐small cell lung cancer. Anticancer Res. 2021;41(2):869‐876. 33517292 10.21873/anticanres.14839

[254] He Y , Zhang X , Zhu M , et al. Soluble PD‐L1: a potential dynamic predictive biomarker for immunotherapy in patients with proficient mismatch repair colorectal cancer. J Transl Med. 2023;21(1):25. 36639643 10.1186/s12967-023-03879-0 PMC9837921

[255] Tian X , Xu WH , Xu FJ , et al. Identification of prognostic biomarkers in papillary renal cell carcinoma and PTTG1 may serve as a biomarker for predicting immunotherapy response. Ann Med. 2022;54(1):211‐226. 35037540 10.1080/07853890.2021.2011956 PMC8765283

[256] Chang P , Chen S , Chang X , Zhu J , Tang Q , Ma L . EXTL3 could serve as a potential biomarker of prognosis and immunotherapy for prostate cancer and its potential mechanisms. Eur J Med Res. 2022;27(1):115. 35818069 10.1186/s40001-022-00740-w PMC9275153

[257] Chen L , Zhou Q , Liu J , Zhang W . CTNNB1 alternation is a potential biomarker for immunotherapy prognosis in patients with hepatocellular carcinoma. Front Immunol. 2021;12:759565. 34777372 10.3389/fimmu.2021.759565 PMC8581472

[258] Deng J , Ma X , Ni Y , et al. Identification of CXCL5 expression as a predictive biomarker associated with response and prognosis of immunotherapy in patients with non‐small cell lung cancer. Cancer Med. 2022;11(8):1787‐1795. 35150082 10.1002/cam4.4567 PMC9041069

[259] Li B , Zhang G , Xu X . APC mutation correlated with poor response of immunotherapy in colon cancer. BMC Gastroenterol. 2023;23(1):95. 36977982 10.1186/s12876-023-02725-3 PMC10053134

[260] Li H , Sun X , Zhao Y , et al. Pan‐cancer analysis of TASL: a novel immune infiltration‐related biomarker for tumor prognosis and immunotherapy response prediction. BMC Cancer. 2023;23(1):528. 37296415 10.1186/s12885-023-11015-w PMC10251564

[261] Dai Y , Qiang W , Lin K , Gui Y , Lan X , Wang D . An immune‐related gene signature for predicting survival and immunotherapy efficacy in hepatocellular carcinoma. Cancer Immunol Immunother. 2021;70(4):967‐979. 33089373 10.1007/s00262-020-02743-0 PMC10992402

[262] Pan D , Hu AY , Antonia SJ , Li CY . A gene mutation signature predicting immunotherapy benefits in patients with NSCLC. J Thorac Oncol. 2021;16(3):419‐427. 33307194 10.1016/j.jtho.2020.11.021 PMC7920921

[263] Zhang Y , Vu T , Palmer DC , et al. A T cell resilience model associated with response to immunotherapy in multiple tumor types. Nat Med. 2022;28(7):1421‐1431. 35501486 10.1038/s41591-022-01799-y PMC9406236

[264] Zhang Y , Wang Q , Yang WK , et al. Development of an immune‐related prognostic biomarker for triple‐negative breast cancer. Ann Med. 2022;54(1):1212‐1220. 35481432 10.1080/07853890.2022.2067894 PMC9068007

[265] Ghiringhelli F , Bibeau F , Greillier L , et al. Immunoscore immune checkpoint using spatial quantitative analysis of CD8 and PD‐L1 markers is predictive of the efficacy of anti‐ PD1/PD‐L1 immunotherapy in non‐small cell lung cancer. EBioMedicine. 2023;92:104633. 37244159 10.1016/j.ebiom.2023.104633 PMC10232659

[266] Tsakiroglou AM , Fergie M , Oguejiofor K , et al. Spatial proximity between T and PD‐L1 expressing cells as a prognostic biomarker for oropharyngeal squamous cell carcinoma. Br J Cancer. 2020;122(4):539‐544. 31806878 10.1038/s41416-019-0634-z PMC7028988

[267] Zhang Z , Wang ZX , Chen YX , et al. Integrated analysis of single‐cell and bulk RNA sequencing data reveals a pan‐cancer stemness signature predicting immunotherapy response. Genome Med. 2022;14(1):45. 35488273 10.1186/s13073-022-01050-w PMC9052621

[268] Zhang HC , Deng SH , Pi YN , et al. Identification and validation in a novel quantification system of ferroptosis patterns for the prediction of prognosis and immunotherapy response in left‐ and right‐sided colon cancer. Front Immunol. 2022;13:855849. 35444656 10.3389/fimmu.2022.855849 PMC9014300

[269] Tang B , Yan R , Zhu J , et al. Integrative analysis of the molecular mechanisms, immunological features and immunotherapy response of ferroptosis regulators across 33 cancer types. Int J Biol Sci. 2022;18(1):180‐198. 34975326 10.7150/ijbs.64654 PMC8692154

[270] Zhou M , Zhang Z , Bao S , et al. Computational recognition of lncRNA signature of tumor‐infiltrating B lymphocytes with potential implications in prognosis and immunotherapy of bladder cancer. Brief Bioinform. 2021;22(3):bbaa047. 32382761 10.1093/bib/bbaa047

[271] Zhang X , Liang H , Tang Q , Chen H , Guo F . Pyroptosis‐related gene to construct prognostic signature and explore immune microenvironment and immunotherapy biomarkers in bladder cancer. Front Genet. 2022;13:801665. 35846123 10.3389/fgene.2022.801665 PMC9283834

[272] Zeng Z , Liang Y , Shi J , et al. Identification and application of a novel immune‐related lncRNA signature on the prognosis and immunotherapy for lung adenocarcinoma. Diagnostics (Basel). 2022;12(11):2891. 36428951 10.3390/diagnostics12112891 PMC9689875

[273] Chen M , Lu H , Copley SJ , et al. A novel radiogenomics biomarker for predicting treatment response and pneumotoxicity from programmed cell death protein or ligand‐1 inhibition immunotherapy in NSCLC. J Thorac Oncol. 2023;18(6):718‐730. 36773776 10.1016/j.jtho.2023.01.089

[274] Bosenberg M , Liu ET , Yu CI , Palucka K . Mouse models for immuno‐oncology. Trends Cancer. 2023;9(7):578‐590. 37087398 10.1016/j.trecan.2023.03.009

[275] Sato Y , Fu Y , Liu H , Lee MY , Shaw MH . Tumor‐immune profiling of CT‐26 and Colon 26 syngeneic mouse models reveals mechanism of anti‐PD‐1 response. BMC Cancer. 2021;21(1):1222. 34774008 10.1186/s12885-021-08974-3 PMC8590766

[276] Denis M , Grasselly C , Choffour PA , et al. In vivo syngeneic tumor models with acquired resistance to anti‐PD‐1/PD‐L1 therapies. Cancer Immunol Res. 2022;10(8):1013‐1027. 35679518 10.1158/2326-6066.CIR-21-0802

[277] Katuwal NB , Park N , Pandey K , et al. Preclinical platform using a triple‐negative breast cancer syngeneic murine model to evaluate immune checkpoint inhibitors. Anticancer Res. 2023;43(1):85‐95. 36585194 10.21873/anticanres.16137

[278] Ghosh S , He X , Huang WC , Lovell JF . Immune checkpoint blockade enhances chemophototherapy in a syngeneic pancreatic tumor model. APL Bioeng. 2022;6(3):036105. 36164594 10.1063/5.0099811 PMC9509203

[279] Dabagian H , Taghvaee T , Martorano P , et al. PARP targeted alpha‐particle therapy enhances response to PD‐1 immune‐checkpoint blockade in a syngeneic mouse model of glioblastoma. ACS Pharmacol Transl Sci. 2021;4(1):344‐351. 33615184 10.1021/acsptsci.0c00206 PMC7887847

[280] Kim H , Kim M , Im SK , Fang S . Mouse Cre‐LoxP system: general principles to determine tissue‐specific roles of target genes. Lab Anim Res. 2018;34(4):147‐159. 30671100 10.5625/lar.2018.34.4.147 PMC6333611

[281] Takada I , Hidano S , Takahashi S , et al. Transcriptional coregulator Ess2 controls survival of post‐thymic CD4(+) T cells through the Myc and IL‐7 signaling pathways. J Biol Chem. 2022;298(9):102342. 35933014 10.1016/j.jbc.2022.102342 PMC9436822

[282] Zhong H , Lu W , Tang Y , et al. SOX9 drives KRAS‐induced lung adenocarcinoma progression and suppresses anti‐tumor immunity. Oncogene. 2023;42(27):2183‐2194. 37258742 10.1038/s41388-023-02715-5 PMC11809655

[283] Galvani E , Mundra PA , Valpione S , et al. Stroma remodeling and reduced cell division define durable response to PD‐1 blockade in melanoma. Nat Commun. 2020;11(1):853. 32051401 10.1038/s41467-020-14632-2 PMC7015935

[284] Chuprin J , Buettner H , Seedhom MO , et al. Humanized mouse models for immuno‐oncology research. Nat Rev Clin Oncol. 2023;20(3):192‐206. 36635480 10.1038/s41571-022-00721-2 PMC10593256

[285] Pearson T , Greiner DL , Shultz LD . Creation of “humanized” mice to study human immunity. Curr Protoc Immunol. 2008;Chapter 15:15.21.11‐15.21.21. 10.1002/0471142735.im1521s81 PMC3023233 18491294

[286] Cheng H , Zheng Z , Cheng T . New paradigms on hematopoietic stem cell differentiation. Protein Cell. 2020;11(1):34‐44. 31201709 10.1007/s13238-019-0633-0 PMC6949320

[287] McCune JM , Namikawa R , Kaneshima H , Shultz LD , Lieberman M , Weissman IL . The SCID‐hu mouse: murine model for the analysis of human hematolymphoid differentiation and function. Science. 1988;241(4873):1632‐1639. 2971269 10.1126/science.241.4873.1632

[288] Phung SK , Miller JS , Felices M . Bi‐specific and tri‐specific NK cell engagers: the new avenue of targeted NK cell immunotherapy. Mol Diagn Ther. 2021;25(5):577‐592. 34327614 10.1007/s40291-021-00550-6

[289] Mhaidly R , Verhoeyen E . Humanized mice are precious tools for preclinical evaluation of CAR T and CAR NK cell therapies. Cancers (Basel). 2020;12(7):1915. 32679920 10.3390/cancers12071915 PMC7409195

[290] Leclercq G , Haegel H , Toso A , et al. JAK and mTOR inhibitors prevent cytokine release while retaining T cell bispecific antibody in vivo efficacy. J Immunother Cancer. 2022;10(1):e003766. 35064010 10.1136/jitc-2021-003766 PMC8785208

[291] Wang M , Yao LC , Cheng M , et al. Humanized mice in studying efficacy and mechanisms of PD‐1‐targeted cancer immunotherapy. Faseb j. 2018;32(3):1537‐1549. 29146734 10.1096/fj.201700740R PMC5892726

[292] Oswald E , Bug D , Grote A , et al. Immune cell infiltration pattern in non‐small cell lung cancer PDX models is a model immanent feature and correlates with a distinct molecular and phenotypic make‐up. J Immunother Cancer. 2022;10(4):e004412. 35483746 10.1136/jitc-2021-004412 PMC9052060

[293] Qin H , Yang L , Chukinas JA , et al. Systematic preclinical evaluation of CD33‐directed chimeric antigen receptor T cell immunotherapy for acute myeloid leukemia defines optimized construct design. J Immunother Cancer. 2021;9(9):e003149. 34531250 10.1136/jitc-2021-003149 PMC8449984

[294] Gambacorta V , Beretta S , Ciccimarra M , et al. Integrated multiomic profiling identifies the epigenetic regulator PRC2 as a therapeutic target to counteract leukemia immune escape and relapse. Cancer Discov. 2022;12(6):1449‐1461. 35255120 10.1158/2159-8290.CD-21-0980 PMC9394393

[295] Seymour L , Bogaerts J , Perrone A , et al. iRECIST: guidelines for response criteria for use in trials testing immunotherapeutics. Lancet Oncol. 2017;18(3):e143‐e152. 28271869 10.1016/S1470-2045(17)30074-8 PMC5648544

[296] Vaseq R , Sharma A , Li Y , Schmidt‐Wolf IGH . Revising the landscape of cytokine‐induced killer cell therapy in lung cancer: focus on immune checkpoint inhibitors. International Journal of Molecular Sciences. 2023;24(6):5626. 36982701 10.3390/ijms24065626 PMC10054817

[297] Mohsenzadegan M , Peng RW , Roudi R . Dendritic cell/cytokine‐induced killer cell‐based immunotherapy in lung cancer: what we know and future landscape. J Cell Physiol. 2020;235(1):74‐86. 31222740 10.1002/jcp.28977

[298] Zhang L , Yang X , Sun Z , et al. Dendritic cell vaccine and cytokine‐induced killer cell therapy for the treatment of advanced non‐small cell lung cancer. Oncol Lett. 2016;11(4):2605‐2610. 27073525 10.3892/ol.2016.4273 PMC4812113

[299] Zhou L , Xiong Y , Wang Y , et al. A phase IB trial of autologous cytokine‐induced killer cells in combination with sintilimab, monoclonal antibody against programmed cell death‐1, plus chemotherapy in patients with advanced non‐small‐cell lung cancer. Clin Lung Cancer. 2022;23(8):709‐719. 35995696 10.1016/j.cllc.2022.07.009

